# Fecal Microbiota Transplantation Reshapes the Physiological Function of the Intestine in Antibiotic-Treated Specific Pathogen-Free Birds

**DOI:** 10.3389/fimmu.2022.884615

**Published:** 2022-06-23

**Authors:** Peng Li, Mingkun Gao, Bochen Song, Yan Liu, Shaojia Yan, Jiaqi Lei, Yizhu Zhao, Guang Li, Tahir Mahmood, Zengpeng Lv, Yongfei Hu, Yuming Guo

**Affiliations:** State Key Laboratory of Animal Nutrition, College of Animal Science and Technology, China Agricultural University, Beijing, China

**Keywords:** antibiotic-treated, bacteria, bird, intestine, fecal microbiota transplantation

## Abstract

The topic about the interactions between host and intestinal microbiota has already caught the attention of many scholars. However, there is still a lack of systematic reports on the relationship between the intestinal flora and intestinal physiology of birds. Thus, this study was designed to investigate it. Antibiotic-treated specific pathogen-free (SPF) bird were used to construct an intestinal bacteria-free bird (IBF) model, and then, the differences in intestinal absorption, barrier, immune, antioxidant and metabolic functions between IBF and bacteria-bearing birds were studied. To gain further insight, the whole intestinal flora of bacteria-bearing birds was transplanted into the intestines of IBF birds to study the remodeling effect of fecal microbiota transplantation (FMT) on the intestinal physiology of IBF birds. The results showed that compared with bacteria-bearing birds, IBF birds had a lighter body weight and weaker intestinal absorption, antioxidant, barrier, immune and metabolic functions. Interestingly, FMT contributed to reshaping the abovementioned physiological functions of the intestines of IBF birds. In conclusion, the intestinal flora plays an important role in regulating the physiological functions of the intestine.

## Introduction

The intestine is not only involved in absorbing nutrients in the diet, but also protects the host from infection by pathogenic microorganisms. In addition, the intestine is considered to be the largest immune organ (1). A healthy intestine is one that is equipped with a complete barrier structure, powerful absorption and immune functions, and a healthy microbial population (2). The intestinal flora plays an extremely important role in maintaining intestinal health. Some scholars believe that by regulating the physiological functions of the intestine, such as the absorption and transport of nutrients, immunity, and the secretion of hormones, the intestinal flora establishes a connection circuit with the brain, which regulates the emotions and behaviors of the host (3, 4). The influence of the intestinal flora on the immune function is particularly important. A study suggested that 80% of the immune response in the intestine was induced by the intestinal flora (5). Simultaneously, the genetic factors and health status of the host also affect the composition of the intestinal flora (6). The cross-talk between the intestinal bacteria and the intestine is closely related to the health of the host. The mechanism is extremely complex, and that complexity is precisely what has caught the attention of scholars.

**Graphical Abstract:**
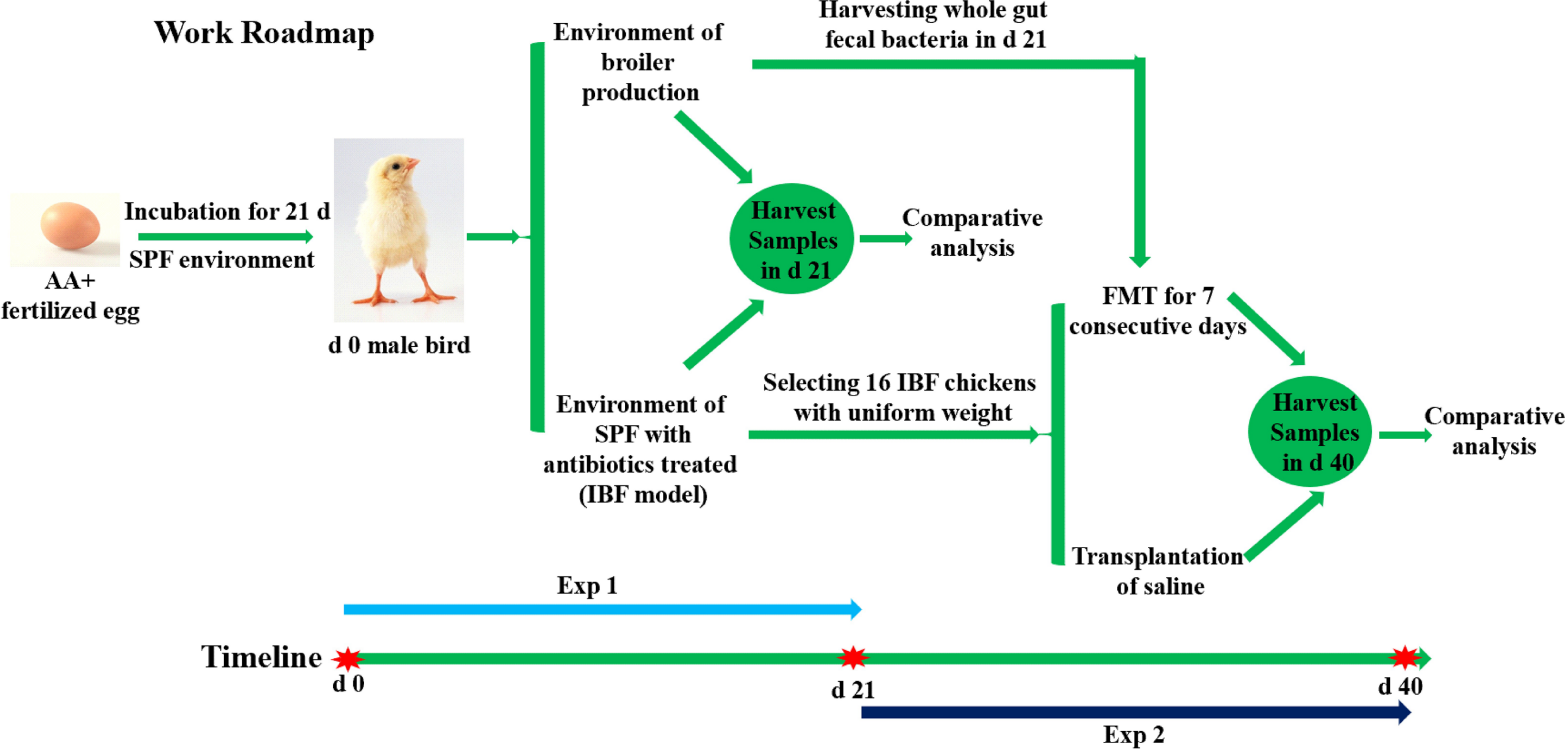


Specific pathogen-free (SPF) and germ-free animal models are considered to be effective tools for studying the relationship between the intestinal flora and host. A study suggested that cecal swelling, intestinal wall atrophy, and decreased intestinal macrophage counts were typical characteristics of sterile mice (7). Based on the SPF bird model, studies found the critical time period for the maturation and establishment of the intestinal flora was 14- 28 days of age (8), and *Lactobacillus plantarum* contributed to alleviating necrotizing enteritis induced by *Clostridium perfringens* (9). Additionally, allicin has been shown to play a potential role against avian reticuloendothelial virus (REV) by blocking the ERK/MAPK pathway (10). The breeding of germ-free animals is difficult, and the breeding conditions are harsh. Therefore, some scholars have used antibiotics to treat SPF mice in the early stages of life, and found that the intestines of the mice were almost sterile (11). Many scholars hypothesized that antibiotic-treated SPF animals could be used for germ-free animal models (12). Fecal microbiota transplantation (FMT) technology is an effective method to study the effects of specific intestinal floras on the host. Studies suggested that FMT was useful for the intestinal development of newborn birds (13), the egg-laying performance of law-laying hens (14), and reducing the difference in the structure of the intestinal flora in the newborn birds (15). In addition, FMT could prevent birds from the developing infection with *Salmonella* (16), and relieve the intestinal inflammation in mice (17). Although some studies about FMT conducted on SPF birds provide useful information, the work is very scarce. Moreover, there remains a lack of systematic reports on the difference in intestinal physiology between germ-free and bacteria-bearing birds.

In the present study, an intestinal bacteria-free bird (IBF) model was constructed by using a combination of antibiotics to treat the intestinal flora of SPF birds in the early stages of life. Then, the differences in intestinal absorption, barrier, antioxidant, immune and metabolic functions between IBF and bacteria-bearing birds were systematically evaluated. In addition, based on the IBF model, the whole intestinal fecal bacteria of bacteria-bearing birds were transplanted into IBF birds, and the effect of FMT on the intestinal physiology of IBF birds was studied. This study aimed to reveal the relationship between the resident bacteria in the intestine and the intestinal physiological function of birds.

## Materials and Methods

### Animal and Diet

The animal experiment was carried out at the Poultry Experiment Base of China Agricultural University (Hebei, China). 410 SPF fertilized AA+ eggs were placed in a SPF hatching room for 21 days of incubation. After the newborn birds were sexed, sixty healthy male birds with uniform weight were selected for follow-up tests. The birds were equally divided into three groups, with 20 birds in each. One group of birds was fed in a normal breeding environment with bacteria (Control), and birds in the other two groups were reared in two isolation barrier systems (Tianjin Jinghang Purification Air Conditioning Company, China) to construct the intestinal bacteria-free bird (IBF) model. The ration formula was formulated based on the nutritional requirements standard of Chinese broilers (NY/T33-2004) (**Table 1**). After the feed was prepared, it was sterilized by radiation (cobalt source, 25K, China Institute of Atomic Energy, Beijing). The drinking water of IBF birds was sterilized (121°C, 103.4 kPa, 15 min), and an antibiotic combination (1 g/L ampicillin, 1 g/L metronidazole, 1 g/L neomycin, and 0.5 g/L vancomycin) was added to the water. The birds in the control group received drinking water without antibiotics. Additionally, all birds had free access to feed, and the cage size, temperature, and lighting conditions were controlled uniformly. At the end of 21 days, 10 birds with uniform body weight from the control group and one group of IBF birds were selected to fast for 8 hours, and then, a 10% D-xylose solution (1 mL/kg BW) was administered orally. One hour later, blood was collected from the underwing vein, and then, these birds were injected intravenously with 50 mg/kg BW sodium pentobarbital, and quickly slaughtered after anesthesia to harvest samples for analysis.

**Table 1:** Test diet composition and nutrition level (air-dry basis).

Ingredients	Contents(%)	Nutritional parameters	Levels^c^
Corn (7.8% pro)	62.644	ME MC/kg	3.016
Dephenolized cottonseed protein (50% pro)	16.200	Crude protein %	21.621
corn gluten meal (51.3% pro)	13.800	Lysine%	1.268
Corn oil	2.000	Methionine%	0.634
CaHPO4	1.980	Calcium %	1.160
Limestone powder	1.100	Total phosphorus %	0.822
L-Lysine HCl (78%)	0.790	Available phosphorus %	0.463
NaCl	0.350	Methionine+Cystine %	0.954
Trace minerals^b^	0.300	Threonine %	0.844
Choline chloride (50%)	0.300	Tryptophan %	0.246
DL-Methionine	0.250		
Threonine	0.140		
Tryptophan	0.080		
Sandaquin	0.030		
multi-vitamins^a^	0.020		
Phytase-5000	0.016		
Total	100		

^a^Vitamin premix (provided per kilogram of feed) the following substances: vitamin A, 12,500 IU; vitamin D3, 2,500 IU; vitamin K3, 2.65 mg; vitamin B1, 2 mg; vitamin B2, 6 mg; vitamin B12, 0.025 mg; vitamin E, 30 IU; biotin, 0.0325 mg; folic acid, 1.25 mg; pantothenic acid, 12 mg; niacin, 50 mg. ^b^Trace element premix (provided per kilogram of feed) the following substances: copper, 8 mg; zinc, 75 mg; iron, 80 mg; manganese, 100 mg; selenium, 0.15 mg; iodine, 0.35 mg. ^c^Calculated value based on the analysis of experimental diets.

### Fecal Microbiota Transplantation

At the end of 21 days, the remaining 10 birds in the control group were slaughtered to obtain whole intestinal chyme to prepare a fecal bacterial suspension. Briefly, the whole intestinal chyme was collected and placed into a 500 mL beaker, and 2 times the volume of normal saline was added and mixed. The mixed liquid was passed through 10-, 18-, 35-, and 60- mesh sieves, and the last filtrate was passed through a 60- mesh sieve three times. The filtrate was centrifuged at 6,000×g for 15 min at 4°C, the pellet was resuspended in sterile normal saline containing 20% glycerol. The prepared bacterial suspension was placed in a refrigerator at 4°C. Sixteen birds with uniform body weight from the remaining IBF birds described above were selected and randomly divided into two treatment groups, with eight birds each. The birds in the FMT group (IBF-FMT) were treated by fecal bacterial transplantation for one week (1 mL/day per bird, the bacterial solution concentration was 1×10^8^ CFU/mL), and the birds in the IBF group (IBF-CTR) were given an equal volume of normal saline. Two weeks after the end of FMT, all birds were selected to fasted for 8 hours, and then, the blood and samples were collected according to the method described above. The animal testing procedure was described in the Work Roadmap (**Graphical Abstract**).

### Serum D-Xylose, DAO, and Cytokine Levels

The blood was centrifuged at 3000 ×g and 4°C for 15 min to separate the serum for later use. The Kits purchased from Nanjing Jiancheng Institute of Biological Engineering (Jiangsu, China) were used to determine the levels of D-xylose and DAO in serum. ELISA kits (IDEXX Laboratories Inc., Weatbrook, Maine, USA) were used to analyze the serum levels of TNF-α, IL-10, IL-1β, IL-4 and IFN-γ.

### Intestinal Morphology, sIgA and Antioxidant Related Enzymes Levels

Sections of the jejunum and ileum approximately 1 cm in length were collected and suspended in a 4% paraformaldehyde solution, and then, the intestinal tissues were stained with periodic acid-Schiff to prepare sections. The method of Wagner et al. (1999) (18) was used to measure the height of intestinal villi (VH) and the depth of crypts (CD), and the ratio of the VH to CD was calculated. At the same time, the number of goblet cells on 100 μm of villi was counted. A tissue homogenate was prepared at a ratio of the weight of the ileal mucosa sample to the volume of physiological saline = 1:9, and then centrifuged at 3000 ×g and 4°C for 15 min to separate the supernatant for later use. A chicken sIgA ELISA kit (Bethyl Laboratories Inc., Montgomery, TX, USA) was used to detect the level of ileal sIgA. Kits purchased from Nanjing Jiancheng Institute of Biological Engineering were used to determine the contents of superoxide dismutase (SOD), total antioxidant capacity (T-AOC), and malondialdehyde (MDA) in the ileum.

### Lymphocyte Analysis of the Ileum

An approximately 3 cm segment of the ileum was taken 1 cm behind the yolk antrum, after the chyme was washed out the intestine was cut into a muddy rough shape in a prechilled calcium and magnesium-free D-Hank’s solution. The treated intestine was moved into a 50 mL centrifuge tube, and five milliliters of separation solution (D-Hank’s solution with 5% FCS, 1 mmol/L DTT, 10 mmol/L HEPES, and 2 mmol/L EDTA) was added into it and shaken for 15 min at 250 r/min and 37°C. After that, the mixture was passed through a 200-mesh cell sieve, the cells on the sieve were collected in a new 50 mL centrifuge tube. Five milliliters of digestion solution (D-Hank’s without calcium and magnesium supplemented with 5% FCS, 0.15% collagenase VIII, and 100 KU/L DNase I) was added to the centrifuge tube and shaken for 45 min at 250 r/min and 37°C. The mixture was passed through a 300-mesh cell sieve, and the filtrate was collected in a 7 mL centrifuge tube. The mixture was centrifuged at 4°C and 400 × g for 10 min to harvest cells and resuspend it into 2 mL of RPMI-1640. Next, 3.3 mL of separation solution was added to a clean 10 mL centrifuge tube, and the cell suspension was carefully transferred to the surface of the separation liquid. The mixture was centrifuged at 4°C and 3000 × g for 30 min, and the white blood cell layer was carefully transferred into a clean 10 mL centrifuge tube. 2 mL of red blood cell lysate was added to the tube and incubated for 5 min. The mixture was centrifuged at 4°C and 3000 × g for 10 min, and 3 mL of D-Hank’s solution was added to the tube to resuspend the cells. After repeating the above centrifugation step, the supernatant was discarded, the cells were resuspended in 2 mL of RPMI-1640, and then, the cell concentration was adjusted to 1×10^7^ cells/mL. According to the method of Li, et al. (19), the percentages of Bu-1^+^, macrophage, CD3^+^, CD4^+^, CD8^+^ lymphocytes were detected and subsequently calculated. The result is expressed as a percentage.

### Gene Expression Level

The jejunum and ileum were collected in RNase-free cryotubes, quickly put into liquid nitrogen, and then stored at -80°C. A 100 mg sample was added to a 1.5 mL centrifuge tube with 1 mL of TRIzol (Invitrogen Life Technologies, Carlsbad, USA) extraction solution, and then, the total RNA was extracted according to the kit instructions (Takara, Dalian, China). After the purity of the total RNA was determined, reverse transcription was performed using an M-MLV cDNA kit (Invitrogen Life Technologies). The reverse transcription product was diluted 1:1 and then subjected to real-time polymerase chain reaction (RT-PCR). Briefly, RT-PCR analysis of gene expression was performed using the primers listed in **Supplementary Table 2**, and SYBR^®^ Premix Ex Taq™ (Takara, Dalian, China) on an Applied Biosystems 7500 Fast Real-Time PCR System (Foster City, CA, USA). The PCR conditions were as follows: 5°C for 2 min, 95°C for 10 min, and 40 cycles of denaturation at 95°C for 15 sec and renaturation at 60°C for 1 min. Finally, the reaction was terminated at 72°C for 10 min. Amplification products were verified by melting curves, agarose gel electrophoresis, and direct sequencing. The results were analyzed by the cycle threshold (CT) method from Fu et al. (2010) (20).

### Microbial Sequencing and Analysis

The ileal and cecal chyme of the control and the IBF-FMT group were collected and then sequenced and analyzed according to the method described by Zhang et al. (2018) (21). Briefly, a fecal bacterial DNA extraction kit (QIAamp Fast DNA Stool Mini Kit, Qiagen Company, Germany) was used to harvest microbial DNA from ileal and cecal chyme. A NanoDrop 2000 (Thermo Scientific, Waltham, MA, USA) was used to determine the concentration of DNA, and 1% agarose gel electrophoresis was used to assess the purity of DNA in the samples. The common primers 338 F (5’-ACTCCTACGGGAGGCAGCA-3’) and 806 R (5’-GGACTACHVGGGTWTCTAAT-3’) targeting the V3-V4 region of the 16S rDNA gene were used to amplify bacterial DNA, and then, the PCR products were purified, quantified and homogenized to construct a sequencing library. A TruSeq^®^ DNA PCR-free sample preparation kit was used for library construction, and the constructed library was quantified by a Qubit and Q-PCR. After the library was qualified, it was sequenced on a system using a HiSeq2500 PE250. The 16S rRNA gene amplicon sequencing results were submitted to the Sequence Read Archive of the NCBI (accession number: PRJNA810526). Sequencing analysis was completed by Beijing Nuohe Zhiyuan Bio-Information Technology Co., Ltd. QIIME software (Qiime2-2019.7, Nature Biotechnology) was used to generate species abundance tables for different taxonomic levels. Based on OTU analysis, the relative abundances of bacteria at the phylum and genus levels were analyzed, and a column chart of the relative abundances of bacteria was drawn.

### Non-Targeted Metabolomics Research

The ileal chyme was collected and stored at -80°C. The metabolites in chyme were used for metabolome sequencing and analysis according to the method of Lu et al. (2019) (22). Briefly, 0.1 g of sample was added to precooled 80% formaldehyde, mixed, and then incubated at -20°C for 60 min. The mixture was centrifuged at 4°C and 14,000 × g for 20 min, and the supernatant was vacuum-dried. Sixty percent formaldehyde buffer was used to dissolve the dried metabolite particles, and then, LC–MS/MS analysis was performed. A 16-min linear gradient was used to inject the sample into a Hypersil Gold column (100 × 2.1 mm, 1.9 µm; Thermo Fisher Scientific) at a flow rate of 0.3 mL/min. The eluents for positive polarity mode were eluent A (0.1% formic acid in water) and eluent B (methanol). The eluents for negative polarity mode were eluent A (5 mmol/L ammonium acetate, pH 9.0) and eluent B (methanol). The solvent gradient settings were as follows: 2% B for 1.5 min, 2-100% B to 12.0 min, 100% B to 14.0 min, 100-2% B to 14.1 min, and 2% B to 16.0 min. The AQ Exactive HF-X mass spectrometer (Thermo Fisher Scientific) was operated in positive/negative polarity mode, with a spray voltage of 3.2 kV, a capillary temperature of 320°C, a sheath gas flow rate of 35 arb, and an auxiliary gas flow rate of 10 arb.

The original files obtained by mass spectrometry were imported into Compound Discoverer 3.1 (Thermo Fisher Scientific) software for analysis. The sequencing analysis was commissioned by Beijing Nuohe Zhiyuan Biological Information Technology Co., Ltd. After the qualitative and quantitative results for metabolites were obtained, the data were subjected to quality control to ensure the accuracy and reliability of the results. Metabolites were analyzed by the multivariate statistical analysis partial least squares discriminant analysis (PLS-DA) method to reveal the differences in the metabolic patterns of different groups. Hierarchical clustering analysis (HCA) and metabolite correlation analysis were used to reveal the relationships between metabolites. Finally, functional analysis was used to explain the biological significance of metabolites.

### Data Analysis

The independent sample T test in SPSS 23.0 software (SPSS Inc., Chicago, IL) was used to analyze the data. The results were shown as the mean ± standard deviation. *P* < 0.05 was considered a significant difference between groups. GraphPad Prism 8.0 software was used to draw figures.

## Results

### FMT Reshaped the Intestinal Bacteria Structure of IBF Chickens

The fecal PCR electrophoresis bands of the control and IBF-FMT group at 1500 bp were bright, and there were no bands in the IBF and the IBF-CTR group (**Supplementary Figure 1**). Additionally, the results of gram staining of the fecal bacterial suspension were consistent with the PCR results. Although we could not determine whether the intestinal tract of the IBF chickens was absolutely sterile, the number of bacteria was at least extremely low. The results also showed that FMT was successful. The weekly test for environmental bacteria showed that there were no bacteria in the growth environment of the birds during the trial. However, due to improper access management of the staff, there was a white mold on the bacterial culture plate used for the detection of environmental bacteria on the day of sampling at the end of the trial (**Supplementary Figure 2**). We believed that it had no effect on the analysis of the study results.

**Figure 1:**
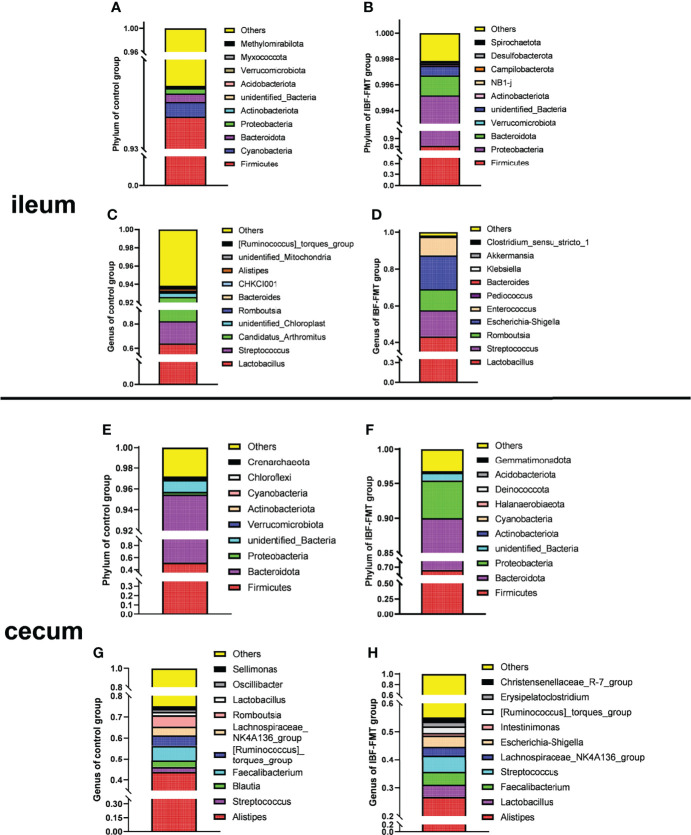
The comparative analysis of intestinal flora structure in control and IBF-FMT group. The main bacteria structure at the phylum and genus level in the ileum (n= 9) and cecum (n= 9) of control group were shown in panels **(A, C)** and panels **(E, G)**, respectively. Based on the IBF bird model, the whole intestinal flora of chickens in the control group were transplanted into the intestine of the chickens of IBF-FMT group, and the main bacteria structure at the phylum and genus level in the ileum (n= 7) and cecum (n= 8) of IBF-FMT group were shown in panels **(B, D)** and panels **(F, H)**, respectively.

The ileal and cecal bacterial composition of the control and the IBF-FMT group were compared and analyzed. In the control group, the ileal bacteria were mainly related to Firmicutes, Bacteroides, Proteobacteria, *Lactobacillus*, and *Staphylococcus* (**Figures 1A, C** **)**. The cecal was dominated by Firmicutes, Bacteroides, Proteobacteria, *Alistipes*, and *Staphylococcus* (**Figures 1E, G** **)**. Previous results showed that there were almost no bacteria in the intestines of IBF birds. Whole intestinal fecal bacteria of the birds in the control group were transplanted into the intestines of IBF birds, and the composition and structure of the main bacteria in the ileum and cecum of the birds in the IBF-FMT group were similar to the control group **(** **Figures 1B, D, F, H** **)**. The results showed that FMT could reshape a complete intestinal bacteria structure in the intestine of IBF birds.

### FMT Reshaped the Intestinal Absorption, Barrier and Antioxidant Functions of IBF Chickens

**Figure 2:**
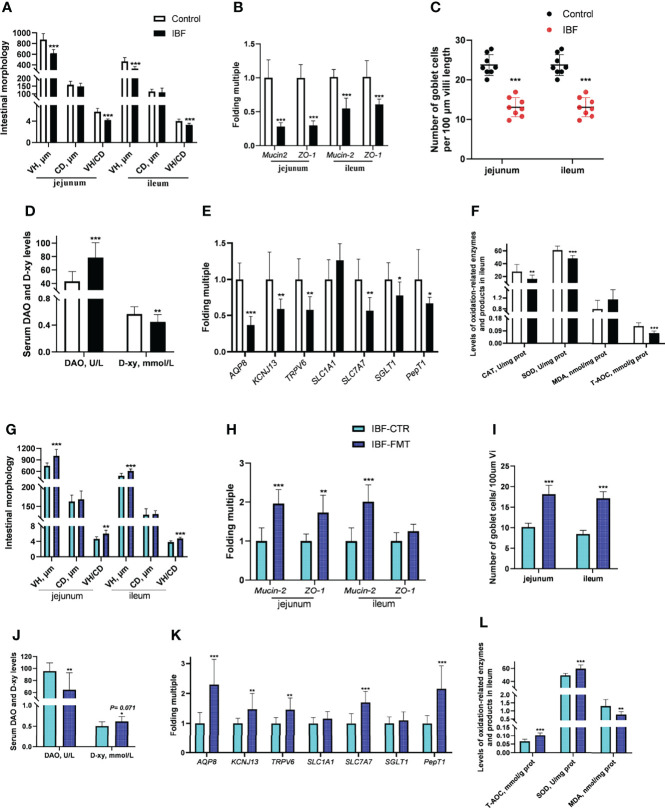
Effects of FMT on intestinal barrier, absorption and antioxidant function in IBF chickens. The results of the comparative study of control and IBF chickens were shown in panels **(A–F)** [as to panels **(A–C)**, n= 8. Additionally, panels **(D–F)**, n= 10], and the comparison results between IBF-CTR and IBF-FMT chickens were shown in panels **(G–L)** (n= 8). Among them, VH= villi height, CD= crypt depth, VH/CD= the ratio of VH to CD, DAO= diamine oxidase, D-xy= D-xylose, CAT= catalase, SOD= superoxide dismutase, MDA= malondialdehyde, and T-AOC= total antioxidant capacity. Additionally, *means that the data tends to be different (0.05< *P*< 0.1), **represents a significant difference (0.001< *P*< 0.05), and ***represents an extremely significant difference (*P*< 0.001), the same below.

Compared with the control group, the IBF chickens had smaller body weight, bursa of fabric and thymus mass index (**Supplementary Figure 3**). The villus height (VH), the ratio of villus height to crypt depth (VH/CD), the number of villus goblet cells, and the mRNA levels of *Mucin-2* and *ZO-1* in the jejunum and ileum of IBF birds were lower than those of the control group (**Figures 2A–C**) (**Supplementary Figure 4**). The gene transcription levels in the jejunum, such as those of aquaporin-8 (*AQP-8*), potassium inwardly rectifying channel subfamily J member 13 (*KCNJ13*), transient receptor potential cation channel subfamily V member 6 (*TRPV6*), and solute carrier family 7 member 7 (*SLC7A7*), of IBF birds were lower than those of birds in the control group, as were the catalase (CAT) and superoxide dismutase (SOD) contents and total antioxidant capacity (T-AOC) in the ileum. In addition, the level of diamine oxidase (DAO) in the serum of IBF birds was higher, and the level of D-xylose was lower than that of the control birds (**Figures 2D–F**). The above evidence showed that the intestinal absorption, barrier and antioxidant functions of IBF birds were weaker than those of the control birds.

To gain more insight, we conducted the FMT on IBF birds, and found FMT elevated the thymus mass index, the VH, VH/CD, number of villus goblet cells, and mRNA level of *Mucin-2* in the jejunum and ileum compared with the IBF-CTR group **(** **Figures 2G–I** **)** (**Supplementary Figure 4**). Additionally, the transcription levels of genes in the jejunum, such as *AQP-8*, *KCNJ13*, *TRPV6*, and *SLC7A7*, and the levels of SOD and T-AOC in the ileum were up-regulated by FMT, as was the serum D-xylose level (**Figures 2J–L**). These findings indicated that FMT contributed to improving the intestinal absorption, barrier and antioxidant functions of IBF chickens. This ability might be the reason why we found that the body weight of the birds in the IBF-FMT group was 130 g higher than that of the birds in the IBF-CTR group (**Supplementary Figure 3**).

### FMT Reshaped the Intestinal Immune Function of IBF Chickens

**Figure 3:**
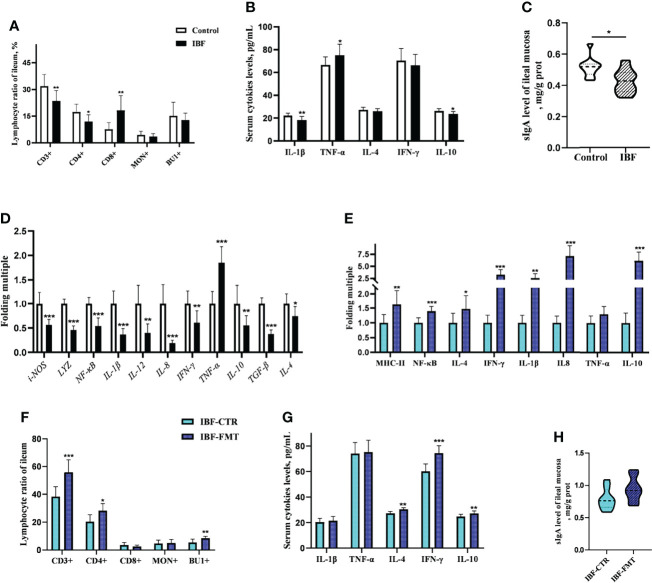
Effects of FMT on intestinal immune function in IBF chickens. The results of the comparative study of control and IBF chickens were shown in panels **(A–D)** (n= 10), and the comparison results between IBF-CTR and IBF-FMT chickens were shown in panels **(E–H)** (n= 8). Among them, * means that the data tends to be different (0.05< P< 0.1), ** represents a significant difference (0.001< P< 0.05), and *** represents an extremely significant difference (P< 0.001), the same below.

The proportion of CD3^+^ and CD4^+^ T cells in the ileum of IBF birds were lower, while the proportion of CD8^+^ T cells was higher than control group **(** **Figure 3A** **)** (**Supplementary Figure 5**). It seemed that CD8^+^ T cells played a key role in immune defense with the removal of intestinal bacteria. Additionally, the levels of serum IL-1β and IL-10, the level of secreted immunoglobulin A (sIgA), and the gene transcription levels in the ileum, such as lysozyme (*LYZ*), *IL-4*, *IL-8*, *IL-10*, interferon-γ (*IFN-γ*), and transforming growth factor β (*TGF-β*) were lower (**Figures 3B–D**). These evidences suggested that the intestinal flora was closely related to the immune function of the intestinal mucosa of host. To gain further insight, the whole intestinal fecal bacteria from chickens in control group was transplanted into the intestine of IBF birds, and found FMT raised the ratio of CD3^+^, CD4^+^ T cells, and B lymphocytes. In addition, the transcription levels of *IL-4*, *IL-8*, *IL-10*, and *IFN-γ* in the ileum, and the levels of serum IL-4, IL-10, and IFN-γ were also increased by FMT (**Figures 3E–H**) (**Supplementary Figure 6**). These evidences illuminated us that FMT was helpful for improving the poor development of intestinal immune function in IBF birds. In fact, this further confirmed the importance of intestinal flora for the immune function of intestine.

### FMT Reshaped the Intestinal Metabolic Function of IBF Chickens

**Figure 4:**
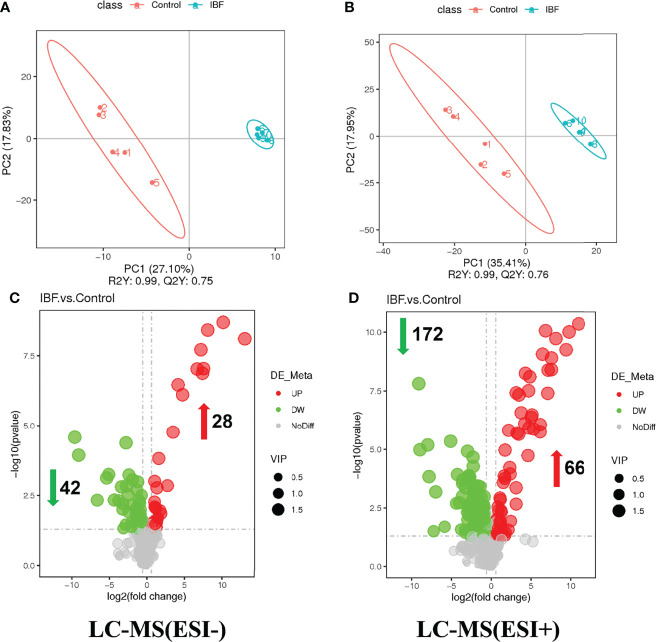
Comparison of ileal chyme metabolome between the birds in IBF and control group. The analysis of panel **(A)** and panel **(B)** were based on the discriminant analysis of partial least squares (PLS-DA). The results in anion mode were showed in panel **(A)** and panel **(C)**, and the results of cation mode were arranged in panel **(B)** and panel **(D)**, n= 5.

**Figure 5:**
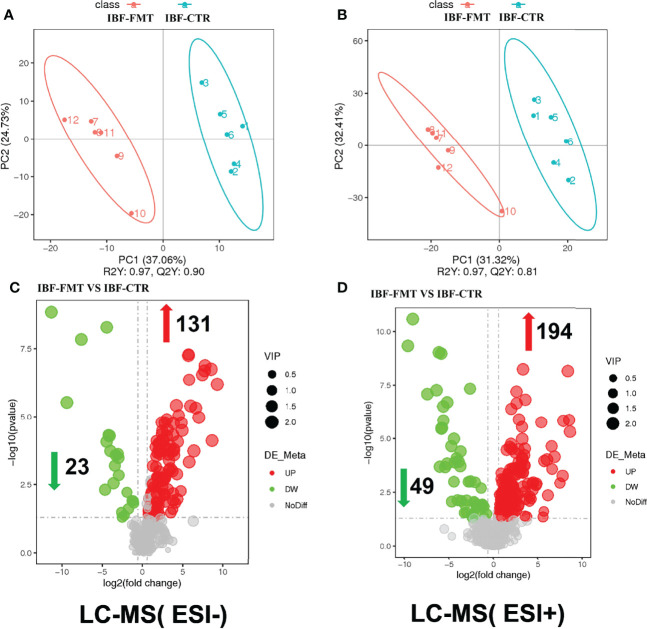
Comparison of ileal chyme metabolome between the birds in IBF-CTR and IBF-FMT group. The analysis of panel **(A)** and panel **(B)** were based on the discriminant analysis of partial least squares (PLS-DA). The results in anion mode were showed in panel **(A)** and panel **(C)**, and the results of cation mode were arranged in panel **(B)** and panel **(D)**, n=6.

**Figure 6:**
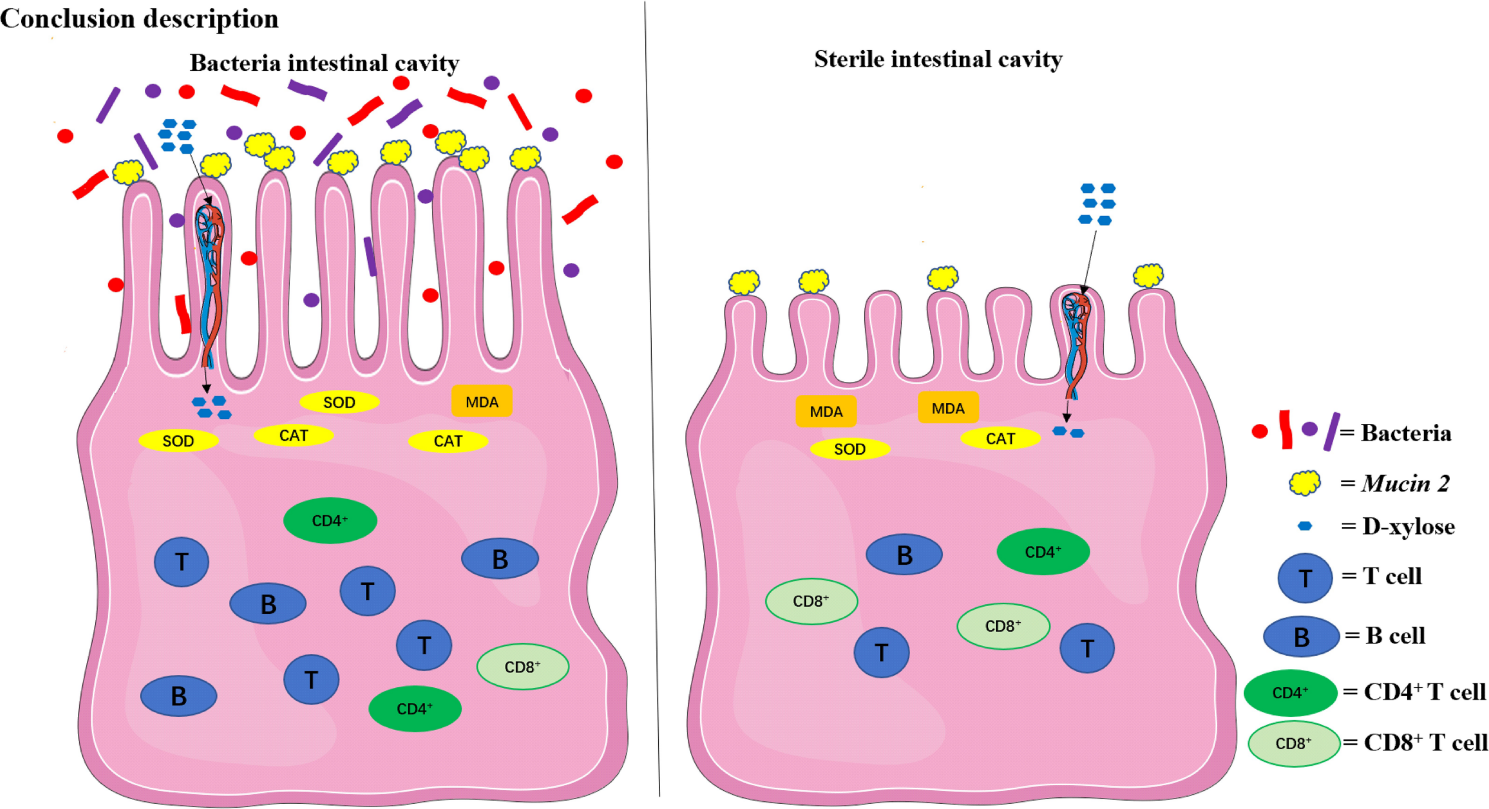
The relationship between the intestinal flora and the function of the intestinal physiology in the present study. The intestinal physiology of the chicken with bacteria in the intestine was described on the left and the IBF on the right. FMT reshaped the physiological function of the intestine in IBF chicken. SOD, superoxide dismutase; CAT, catalase; MDA, malondialdehyde.

The compositions of metabolites in the intestinal chyme of IBF birds and control were different. Specifically, there were 214 metabolites were downregulated, and 94 metabolites were upregulated (**Figure 4**). KEGG analysis showed that these differentially abundant metabolites were mainly enriched in the global and overview maps, the amino acid, vitamin and cofactor, nucleotide, and lipid metabolism pathways. At the same time, differentially abundant metabolites were mainly enriched in the cell membrane transport and protein translation pathways (**Supplementary Figure 7**). With intestinal bacteria cleared, the intestinal metabolic function was severely affected, and these changes involved the metabolism of almost all nutrients. Interestingly, we transplanted the fecal bacteria of the birds in the control group into the intestines of IBF birds and found that the levels of 51 metabolites that were downregulated in the IBF group versus the control group were upregulated by FMT treatment, and that the levels of 18 of the upregulated metabolites were downregulated (**Figure 5**) (**Supplementary Figures 9**, **10**). Additionally, FMT reversed the abovementioned changes in the metabolic pathways in the intestines of IBF birds (**Supplementary Figure 8**). The results demonstrated that bacteria in the intestine participated in the entire process of dietary nutrient metabolism, and that FMT helped reshape the intestinal metabolic function of IBF birds. A schematic representation of the relationship between the intestinal flora and the function of the intestinal physiology was described in **Figure 6**.

## Discussion

The nutrients in the diet are metabolized by intestinal bacteria to produce short-chain fatty acids (SCFAs), functional amino acids, vitamins and other functional substances, which improve the digestion and absorption of nutrients by intestinal epithelial cells and promote intestinal development (23–25). Intestinal bacteria can also directly regulate the absorption of nutrients by intestinal epithelial cells. A study found that intestinal bacteria regulated the transport and absorption of lipids in intestinal epithelial cells by regulating the expression of a circadian transcription factor (NFIL3) (26). In the present study, the mRNA levels of intestinal nutrient transporters in the intestine of IBF birds were lower than those of birds with bacteria in the intestine. The results were similar to the findings of previous studies on mice (7). Some scholars have evaluated the absorption function of the intestine by measuring the absorption capacity of D-xylose in piglets under fasting conditions (27). In our study, the level of serum D-xylose in IBF birds was lower than that in control birds. This evidence indicated that the intestinal absorption function of IBF birds was weak and served as a reason that the body weight of the birds was lighter. Intestinal bacteria are involved in the secretion of mucin and contribute to intestinal barrier function (28), and a high level of DAO in serum is regarded as one of the markers of intestinal barrier damage (7). In the present study, the number of goblet cells and the mRNA level of *Mucin-2* in the intestine of IBF birds were less than those of control birds, and the serum DAO level was higher. As a result, we found that the intestinal morphology of IBF birds was worse than that of the control birds. A study suggested that when the fecal bacteria of birds with high feed conversion efficiency (FCR) were transplanted into the intestines of birds with low FCR, the feed intake and body weight of the birds were increased (29). A study also suggested that FMT was beneficial to the intestinal absorption and barrier functions of birds (30). In this study, we found that FMT improved the intestinal morphology and the mRNA levels of intestinal nutrient transporter genes of IBF birds. Our findings lead us to conclude that FMT could reshape the intestinal absorption and barrier functions of IBF birds.

With antibiotic treatment in early life, the intestines become highly sensitive to stress, and a large number of inflammatory factors and oxygen free radicals are produced accordingly (31). We found that the levels of SOD, CAT and the T-AOC in the intestines of IBF birds were lower than those in the intestines of control birds. This finding demonstrated that intestinal bacteria might play a significant role in improving antioxidant function. Studies have found that FMT could improve the antioxidant function of newborn and weaned piglets (32, 33) and relieve oxidative stress caused by acute lung injury in mice (34). To gain further insight, IBF birds were transplanted with the whole intestinal fecal bacteria of the birds in the control group in this study. Interestingly, FMT raised the level of SOD and the T-AOC in the intestine of IBF birds, and decreased the level of MDA. On the basis of these results, we concluded that intestinal bacteria were essential for the antioxidant capacity of the host.

Intestinal bacteria and their metabolites are necessary not only for immune homeostasis but also for determining the host’s susceptibility to many diseases. The stable structure of the intestinal bacterial community participates in shaping the immune function of the intestinal mucosa. Once the structure of the intestinal flora is destroyed, immune homeostasis becomes unbalanced (2, 35). A study suggested that intestinal bacteria were involved in regulating the maturation and differentiation of CD4^+^ and Treg T cells, and maintaining intestinal immune homeostasis (36). In the present study, the number of T and B lymphocytes in the ileum of IBF birds was lower than that of control birds. Notably, the proportion of CD4^+^ T cells in the ileum of IBF birds was lower than that of the control birds, while the proportion of CD8^+^ T cells was higher. CD4^+^ T cells are regarded as important “helpers” of the immune system and are involved in maintaining the body’s immune homeostasis. The decrease in the proportion of CD4^+^ T cells indicated that the body was in a state of immunosuppression. CD8^+^ T cells directly participate in killing infected cells, and an increase in their proportion is common in immunosuppression (37). Our findings led us to conclude that IBF birds were in an immunosuppressed state and that CD8^+^ T cells might play an important role in the process of immune defense. Cytokines are involved in regulating the body’s immune homeostasis, and the proper expression of proinflammatory factors such as IL-1β, TNF-α, and IL-6 activates the immune system. Once overexpressed, this cytokine expression causes inflammation (38). Anti-inflammatory factors such as IL-4 and IL-10 participate in immune tolerance and antibody synthesis (39). In the present study, the levels of serum IL-1β, IL-10 and the mRNA levels of ileal cytokines were lower in IBF birds than in control birds. The decrease in the levels of these cytokines seemed to be related to the decrease in the number of intestinal immune cells. A study suggested that intestinal bacteria stimulated the secretion of sIgA by promoting the proliferation of intestinal dendritic cells (40). sIgA helps prevent pathogen colonization of the intestinal mucosa, and sIgA is considered to be the main immune barrier that maintains the homeostasis of the symbiotic flora (41). In the present study, the level of ileal sIgA and the weight index of immune organs in IBF birds were lower than those in control birds. These findings further proved that intestinal bacteria played a critical role in regulating the immune function of the host. This finding was consistent with previous study (1, 5, 42).

Studies have suggested that FMT reshaped local and systemic immune development in sterile mice (43), and relieved intestinal flora disorder and immune stress caused by antibiotic abuse (44, 45). In this study, the fecal bacteria of bacteria-bearing birds were transplanted into IBF birds, and found FMT increased the weight of immune organs and the levels of intestinal immune cells and cytokines in IBF birds. This result indicated that FMT reshaped the immune function of IBF birds. A study suggested that transplantation of the whole intestinal flora helped bacteria from different intestinal segments colonize the corresponding locations (46). The commensal bacteria in the intestine provide colonization resistance against pathogenic bacteria by competing for niches and nutrients and metabolizing bacteriocins, SCFAs and other bactericidal substances. This activity contributes to maintaining the homeostasis of the intestinal environment (47). In the present study, the fecal bacteria from birds with bacteria in the control group were transplanted into the intestines of IBF birds, and it was found that the intestinal bacteria of IBF birds could be shaped into a flora structure that was similar to that of the control birds. This evidence indicated that FMT shaped the structure of the intestinal bacteria of IBF birds. This finding was consistent with a previous study in sterile mice (48). On the basis of our findings, it could be concluded that FMT shaped the immune function of the intestine by reshaping the structure of the intestinal bacterial community.

The genes encoding metabolic enzymes carried by the gut microbiota are far more abundant than those of the host, so the gut microbiota is equipped with powerful metabolic capabilities. Diets and host-derived substrates, such as polysaccharides, bile acids and choline, are independently metabolized by intestinal bacteria on the one hand as well as jointly metabolized by the host in coordination (49, 50). Studies have suggested that intestinal bacteria help maintain the metabolic homeostasis of the host. The imbalance of intestinal bacteria leads to abnormal metabolism of metabolites such as branched-chain amino acids, hormones, vitamins, and SCFAs, which gives rise to the development of host diseases (51). To clarify the specific metabolic pathways that involve intestinal bacteria, the ileum chyme of IBF and bacteria-containing birds was analyzed by metabolomics. The metabolic pathways of almost all nutrients changed as the intestinal bacteria were cleared. Among them, amino acid, vitamin and cofactor, nucleotide, lipid and other metabolic pathways were most enriched. Some scholars believe that carbohydrates in diets fermented by intestinal bacteria mainly produce SCFAs such as butyric acid, acetic acid and propionic acid, which could be used as energy sources by the intestinal epithelial cells of the host and contribute to intestinal immune function (52). SCFAs, long-chain polyunsaturated fatty acids (PUFAs), bile acids and some methylamine-containing substances, such as choline, lecithin and L-carnitine, are considered to be products of intestinal bacteria metabolizing dietary lipids. These substances play an important role in regulating intestinal absorption, barrier, and immune functions (53). Additionally, intestinal bacteria participate in regulating the energy metabolism of the host by regulating the metabolism of vitamins, especially B and K vitamins (54). A study suggested that intestinal bacteria also regulate intestinal physiology by improving the metabolism of amino acids such as aromatic amino acids and branched-chain amino acids (55). In the present study, the abovementioned differentially enriched metabolic pathways were reshaped by FMT. This evidence led us to conclude that intestinal bacteria were involved in the metabolic process of almost all nutrients in the host, helping maintain normal intestinal physiology. Our findings also illuminated us that FMT might reshape the physiology of the host’s intestine by reshaping the structure of the intestinal bacterial community and metabolic pathways.

## Conclusion

Antibiotic treatment of SPF birds in the early stages of life could be used to construct an intestinal bacteria-free bird model. Intestinal bacteria participated in the regulation of intestinal absorption, barrier, antioxidant, immune and metabolic functions. FMT reshaped the physiology of the host’s intestine by reshaping the structure of the intestinal bacterial community and metabolic pathways.

## Data Availability Statement

The original contributions presented in the study are publicly available. This data can be found here: https://www.ncbi.nlm.nih.gov/search/all/?term=PRJNA810526.

## Ethics Statement

The animal study was reviewed and approved by Animal Ethics Committee of China Agricultural University, Beijing, China.

## Author Contributions

YG and PL designed the study, PL wrote the manuscript. PL, MG, YL, BS, SY, JL, YZ, and GL collected and analyzed experimental results. TM, ZL, YH, and YG participated in the writing and revision of the manuscript. All authors contributed to the data interpretation and approved the final version of the manuscript.

## Funding

This project was funded by the China Agriculture Research System program (CARS-41-G11).

## Conflict of Interest

The authors declare that the research was conducted in the absence of any commercial or financial relationships that could be construed as a potential conflict of interest.

## Publisher’s Note

All claims expressed in this article are solely those of the authors and do not necessarily represent those of their affiliated organizations, or those of the publisher, the editors and the reviewers. Any product that may be evaluated in this article, or claim that may be made by its manufacturer, is not guaranteed or endorsed by the publisher.

## Supplementary Material

Supplementary Figure 1 The results of PCR and gram staining about the whole intestinal chyme mixture. According to the standard of China’s sterile animal living environment and fecal specimen testing standards (GB/T 14926.41-2001), six samples of each treatment were randomly selected for PCR analysis. The primer sequence was 27F: 5’-AGAGTTTGATCCTGGCTCAG-3’, 1492R: 5’- TACGGYTACCTTGTTACGACTT-3’. The result was shown above **(A, B)**, and the NC represented the PCR results of RNA-free water. Then, the intestinal chyme of all birds in each treatment were mixed separately, and three samples from the mixture were randomly selected for gram stain observation. The results were shown in **(C–F)**, among them, the gram-positive bacteria were stained purple, and the red ones represented the gram-negative bacteria.

Supplementary Figure 2 The results of sedimentation bacteria in SPF environment. According to the standard of China’s sterile animal living environment and fecal specimen testing standards (GB/T 14926.41-2001), Different corners of the SPF environment were selected for sedimentation bacteria detection every week, and feeding management staff wear isolation equipment for work every day.

Supplementary Figure 3 Effects of FMT on the body weight, organ mass index. The comparison results of control and IBF group were shown in **(A, B)**. The comparison results of IBF-CTR and IBF-FMT group were arranged in **(C, D)**. Among them, * means that the data tends to be different (0.05< *P*< 0.1), ** represents a significant difference (0.001< *P*< 0.05).

Supplementary Figure 4 Effects of FMT on intestinal morphology and the number of goblet cells. The purple dots attached to the intestinal villi represents goblet cells, the picture magnification was 400 times, n= 8.

Supplementary Figure 5 The flow cytometry analysis results of intestinal immune cells between IBF birds and control. Our analysis steps for flow cytometry results were as follows. At first, we use the CD45 ring gate to eliminate the interference of red blood cells. In the gate of CD45^+^, T lymphocytes were labeled with CD3^+^ and their ratios were obtained, and then B lymphocytes and monocytes were labeled with Bu1^+^ and Mon^+^, and their ratios were obtained. In the gate of CD3^+^, the ratios of CD4^+^ and CD8^+^ T cell were obtained, the same below.

Supplementary Figure 6 The flow cytometry analysis results of intestinal immune cells between the birds in IBF-control and IBF-FMT group. The flow cytometry analysis process was the same as **Supplementary Figure 5**.

Supplementary Figure 7 The pathway enrichment of differential metabolites based on KEGG between IBF birds and control. The results in the negative ion mode were arranged on the left, and the results in the positive ion mode were displayed on the other side, n=5.

Supplementary Figure 8 The pathway enrichment of differential metabolites based on KEGG between IBF-CTR birds and IBF-FMT. The results in the negative ion mode were arranged on the left, and the results in the positive ion mode were displayed on the other side, n=6.

Supplementary Figure 9 The differential metabolites of ileal chyme in anion mode between the birds in IBF-CTR and IBF-FMT group. Substances that were up- or down-regulated by IBF compared with the control group were reshaped by FMT, and these substances were marked by red arrows.

Supplementary Figure 10 The differential metabolites of ileal chyme in cation mode between the birds in IBF-CTR and IBF-FMT group. Substances that were up- or down-regulated by IBF compared with the control group were reshaped by FMT, and these substances were marked by red arrows.
