# Supplementary material for: Fecal Microbiota Transplantation Reshapes the Physiological Function of the Intestine in Antibiotic-Treated Specific Pathogen-Free Birds
Source: Front Immunol. 2022 Jun 23;13:884615. doi: 10.3389/fimmu.2022.884615 (PMC9261465; doi:10.3389/fimmu.2022.884615)
Supplement: Supplementary Figure 1 — The results of PCR and gram staining about the whole intestinal chyme mixture. According to the standard of China’s sterile animal living environment and fecal specimen testing standards (GB/T 14926.41-2001), six samples of each treatment were randomly selected for PCR analysis. The primer sequence was 27F: 5’-AGAGTTTGATCCTGGCTCAG-3’, 1492R: 5’- TACGGYTACCTTGTTACGACTT-3’. The result was shown above (A, B), and the NC represented the PCR results of RNA-free water. Then, the intestinal chyme of all birds in each treatment were mixed separately, and three samples from the mixture were randomly selected for gram stain observation. The results were shown in (C–F), among them, the gram-positive bacteria were stained purple, and the red ones represented the gram-negative bacteria. [file DataSheet_1.zip › Supplementatry Figures/Supplementary_Fig legends and Tables.docx]

Supplementary Material

# Supplementary Figures and Tables

## 1.1 Supplementary Figures legend

**Supplementary Fig. 1 The results of PCR and gram staining about the whole intestinal chyme mixture.** According to the standard of China's sterile animal living environment and fecal specimen testing standards (GB/T 14926.41-2001), six samples of each treatment were randomly selected for PCR analysis. The primer sequence was 27F: 5’-AGAGTTTGATCCTGGCTCAG-3’, 1492R: 5’- TACGGYTACCTTGTTACGACTT-3’. The result was shown above A and B, and the NC represented the PCR results of RNA-free water. Then, the intestinal chyme of all birds in each treatment were mixed separately, and three samples from the mixture were randomly selected for gram stain observation. The results were shown in C- F, among them, the gram-positive bacteria were stained purple, and the red ones represented the gram-negative bacteria.

**Supplementary Fig. 2 The results of sedimentation bacteria in SPF environment.** According to the standard of China's sterile animal living environment and fecal specimen testing standards (GB/T 14926.41-2001), Different corners of the SPF environment were selected for sedimentation bacteria detection every week, and feeding management staff wear isolation equipment for work every day.

**Supplementary Fig. 3 Effects of FMT on the body weight, organ mass index.** The comparison results of control and IBF group were shown in Supplementary Fig.3 A and B. The comparison results of IBF-CTR and IBF-FMT group were arranged in Supplementary Fig.3 C and D. Among them, * means that the data tends to be different (0.05< *P*< 0.1), ** represents a significant difference (0.001< *P*< 0.05).

**Supplementary Fig. 4 Effects of FMT on intestinal morphology and the number of goblet cells.** The purple dots attached to the intestinal villi represents goblet cells, the picture magnification was 400 times, n= 8.

**Supplementary Fig. 5 The flow cytometry analysis results of intestinal immune cells between IBF birds and control**. Our analysis steps for flow cytometry results were as follows. At first, we use the CD45 ring gate to eliminate the interference of red blood cells. In the gate of CD45^+^, T lymphocytes were labeled with CD3^+^ and their ratios were obtained, and then B lymphocytes and monocytes were labeled with Bu1^+^ and Mon^+^, and their ratios were obtained. In the gate of CD3^+^, the ratios of CD4^+^ and CD8^+^ T cell were obtained, the same below.

**Supplementary Fig. 6 The flow cytometry analysis results of intestinal immune cells between the birds in IBF-control and IBF-FMT group**. The flow cytometry analysis process was the same as Supplementary fig. 5.

**Supplementary Fig. 7 The pathway enrichment of differential metabolites based on KEGG between IBF birds and control.** The results in the negative ion mode were arranged on the left, and the results in the positive ion mode were displayed on the other side, n= 5.

**Supplementary Fig. 8 The pathway enrichment of differential metabolites based on KEGG between IBF-CTR birds and IBF-FMT.** The results in the negative ion mode were arranged on the left, and the results in the positive ion mode were displayed on the other side, n= 6.

**Supplementary Fig.9 The differential metabolites of ileal chyme in anion mode between the birds in IBF-CTR and IBF-FMT group.** Substances that were up- or down-regulated by IBF compared with the control group were reshaped by FMT, and these substances were marked by red arrows.

**Supplementary Fig.10 The differential metabolites of ileal chyme in cation mode between the birds in IBF-CTR and IBF-FMT group.** Substances that were up- or down-regulated by IBF compared with the control group were reshaped by FMT, and these substances were marked by red arrows.

## 1.2 Supplementary Tables

| **Table 2. List of gene primer sequences ^a^** | | |
| --- | --- | --- |
| **Gene name ^b^** | **Prime sequence（5′-3′）** | **NCBI number** |
| *NF-κB* | F-GTGTGAAGAAACGGGAACTG | NM_205129.1 |
|  | R-GGCACGGTTGTCATAGATGG |  |
| *IL-12* | F-AAGGTGCAGAAGCAGAGGAC | NM_213588.1 |
|  | R-TTGTGTTGCTCTGACTGTTGG |  |
| *IL-8* | F-ATGAACGGCAAGCTTGGAGCTG | NM_205498.2 |
|  | R-TCCAAGCACACCTCTCTTCCATCC |  |
| *IL-4* | F-AGACAAATAACAAAACTGAGC | XM_040646929.1 |
|  | R-TTGGTGGAAGAAGGTACG |  |
| *IFN-γ* | F-AGCTGACGGTGGACCTATTATT | NM_205149.1 |
|  | R-GGCTTTGCGCTGGATTC |  |
| *i-NOS* | F-CCTGTACTGAAGGTGGCTATTGG | NM_204961.1 |
|  | R-AGGCCTGTGAGAGTGTGCAA |  |
| *TNF-α* | F-GAGCGTTGACTTGGCTGTC | XM_040647309.1 |
|  | R-AAGCAACAACCAGCTATGCAC |  |
| *IL-1β* | F-ACTGGGCATCAAGGGCTA | XM_015297469.2 |
|  | R-GGTAGAAGATGAAGCGGGTC |  |
| *TGF-β* | F-TCATCACCAGGACAGCGTTA | NM_001031045.3 |
|  | R-TGTGATGGAGCCATTCATGT |  |
| *Mucin-2* | F-TCCCCTGTTGAGGGAGAACTT | XM_040673077.1 |
|  | R-AGTGGTTGTACCTTCGGTGC |  |
| *AQP-8* | F-CCTTTGGGCCAGCTGTGATA | XM_040684010.1 |
|  | R-CACTTCAGGAACAGGCGGAT |  |
| *KCNJ-13* | F-ACACCACCTGCTCTGAACAC | XM_040679164.1 |
|  | R-TAGAGATCTCCTTAAGGCCACTTG |  |
| *TRPV6* | F-CTGTGCTCACGTCCTCTGTT | XM_040661661.1 |
|  | R-TGTTGCTGTGTGACAGATGGT |  |
| *SLC1A1* | F-TGGCAAGCTGTCTAACCTGG | XM_424930.7 |
|  | R-GCTCGCAAACCAATCTTCCC |  |
| *SLC7A7* | F-CTGGTGTTGGACTCCCTTGT | XM_040665181.1 |
|  | R-TCCGCAGTTTTTCGCTTGTG |  |
| *SGLT1* | F-GATGTGCGGATACCTGAAGC | NM_001293240.1 |
|  | R-AGGGATGCCAACATGACTGA |  |
| *PepT1* | F-TACGCATACTGTCACCATCA | NM_204365.2 |
|  | R-TCCTGAGAACGGACTGTAAT |  |
| *ZO-1* | F-TATGAAGATCGTGCGCCTCC | XM_040680632.1 |
|  | R-GAGGTCTGCCATCGTAGCTC |  |
| *IL-10* | F-CGCTGTCACCGCTTCTTCA | NM_001004414.2 |
|  | R-CGTCTCCTTGATCTGCTTGATG |  |
| *LYZ* | F-CCCAGGCTCCAGGAACCT | NM_205281.1 |
|  | R-CACGCTCGCTGTTATGTCTGA |  |
| *MHC-II* | F-ATAAGGCGTGGGCTCAGTTC | NM_001245061.1 |
|  | R-GAATTCGGGCAGCCTCCATA |  |
| *β-actin* | F-GAGAAATTGTGCGTGACATCA | NM_205518.1 |
|  | R-CCTGAACCTCTCATTGCCA |  |

^a^ Primers designed using Primer Express software (Sangon Biotech, Shanghai, China).

^b^ Abbreviation, *NF-κB*= nuclear transcription factor kappa B, *i-NOS*= inducible-nitric oxide synthase, *IFN-γ*= interferon-gama, *AQP-8*= aquaporin 8, *KCNJ13*= potassium inwardly rectifying channel subfamily J member 13, *TRPV6*= transient receptor potential cation channel, subfamily V member 6, *SLC1A1*= solute carrier family 1 member 1, *SLC7A7*= solute carrier family 1 member 1, *SGLT1*= solute carrier family 5 member 1, *PepT1*= solute carrier family 15 member 1, *TNF-α*= tumor necrosis factor α, *IL-1β, IL-4, IL-8, IL-12,* and *IL-10*= interleukin 1β, 4, 8, 12, and 10, *TGF-β*= transforming growth factor β, *Mucin-2*= mucin family protein 2, *ZO-1* belongs to tight junction proteins, *LYZ*= lysozyme, *MHC-II*= major histocompatibility complex II.
