# Supplementary figures and images for: Fecal Microbiota Transplantation Reshapes the Physiological Function of the Intestine in Antibiotic-Treated Specific Pathogen-Free Birds
Source: Front Immunol. 2022 Jun 23;13:884615. doi: 10.3389/fimmu.2022.884615 (PMC9261465; doi:10.3389/fimmu.2022.884615)

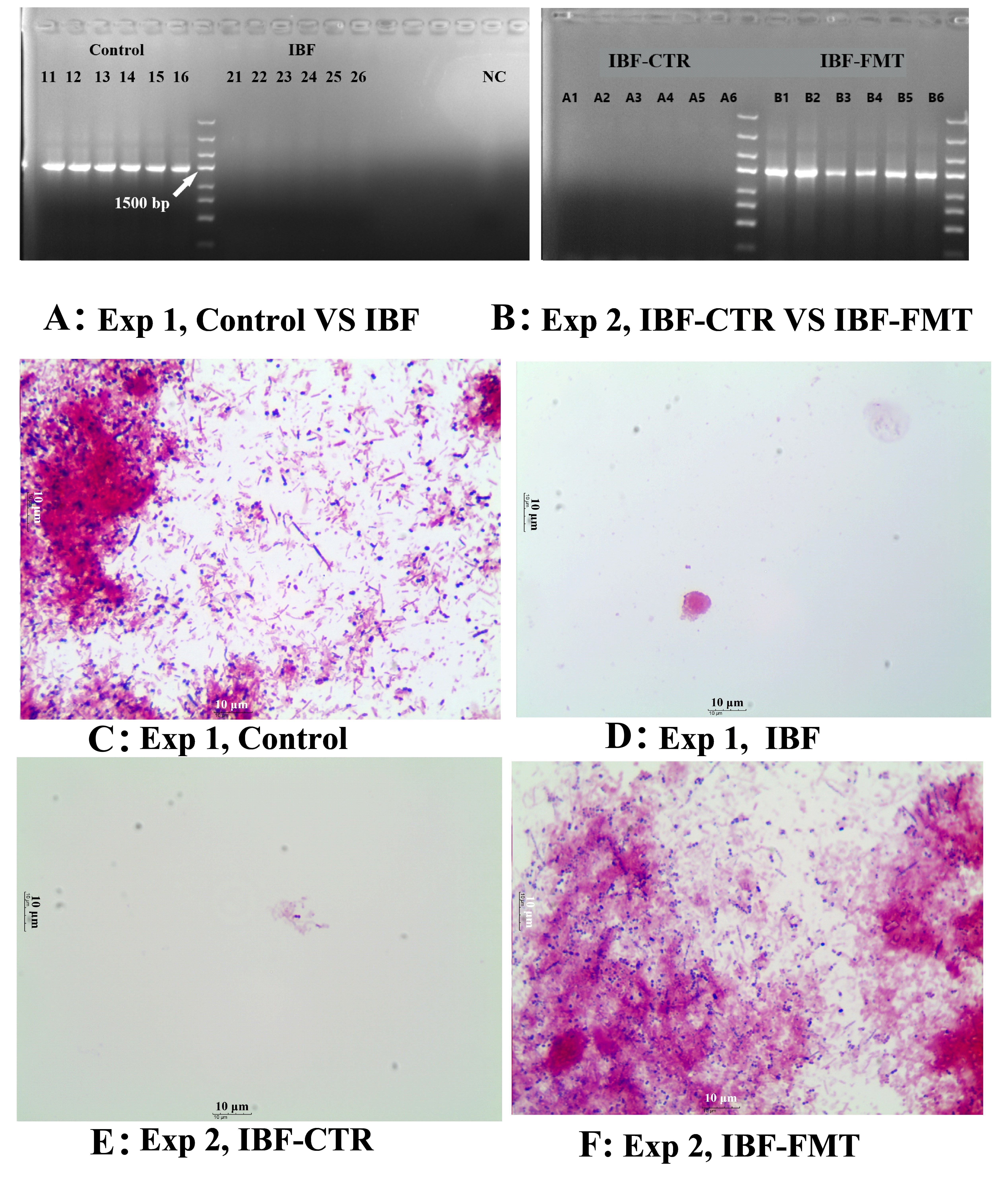

Supplement: Supplementary Figure 1 — The results of PCR and gram staining about the whole intestinal chyme mixture. According to the standard of China’s sterile animal living environment and fecal specimen testing standards (GB/T 14926.41-2001), six samples of each treatment were randomly selected for PCR analysis. The primer sequence was 27F: 5’-AGAGTTTGATCCTGGCTCAG-3’, 1492R: 5’- TACGGYTACCTTGTTACGACTT-3’. The result was shown above (A, B), and the NC represented the PCR results of RNA-free water. Then, the intestinal chyme of all birds in each treatment were mixed separately, and three samples from the mixture were randomly selected for gram stain observation. The results were shown in (C–F), among them, the gram-positive bacteria were stained purple, and the red ones represented the gram-negative bacteria. [file DataSheet_1.zip › Supplementatry Figures/Supplementatry Fig. 1.jpg]

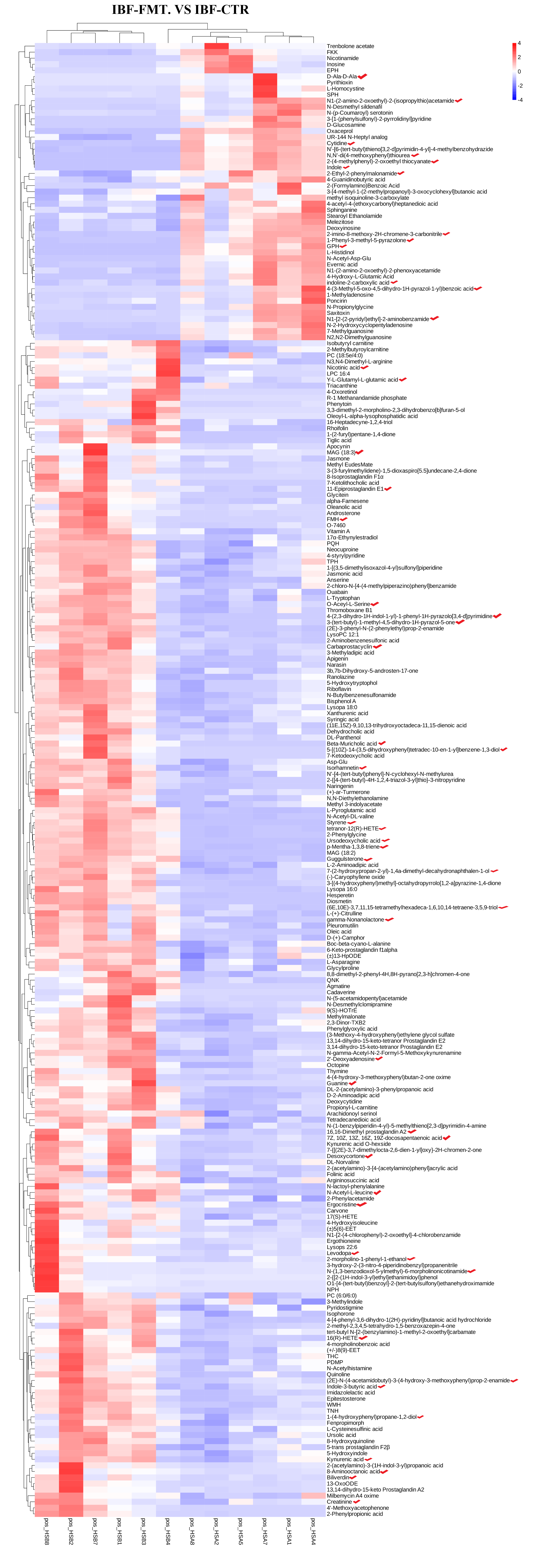

Supplement: Supplementary Figure 1 — The results of PCR and gram staining about the whole intestinal chyme mixture. According to the standard of China’s sterile animal living environment and fecal specimen testing standards (GB/T 14926.41-2001), six samples of each treatment were randomly selected for PCR analysis. The primer sequence was 27F: 5’-AGAGTTTGATCCTGGCTCAG-3’, 1492R: 5’- TACGGYTACCTTGTTACGACTT-3’. The result was shown above (A, B), and the NC represented the PCR results of RNA-free water. Then, the intestinal chyme of all birds in each treatment were mixed separately, and three samples from the mixture were randomly selected for gram stain observation. The results were shown in (C–F), among them, the gram-positive bacteria were stained purple, and the red ones represented the gram-negative bacteria. [file DataSheet_1.zip › Supplementatry Figures/Supplementatry Fig. 10.png]

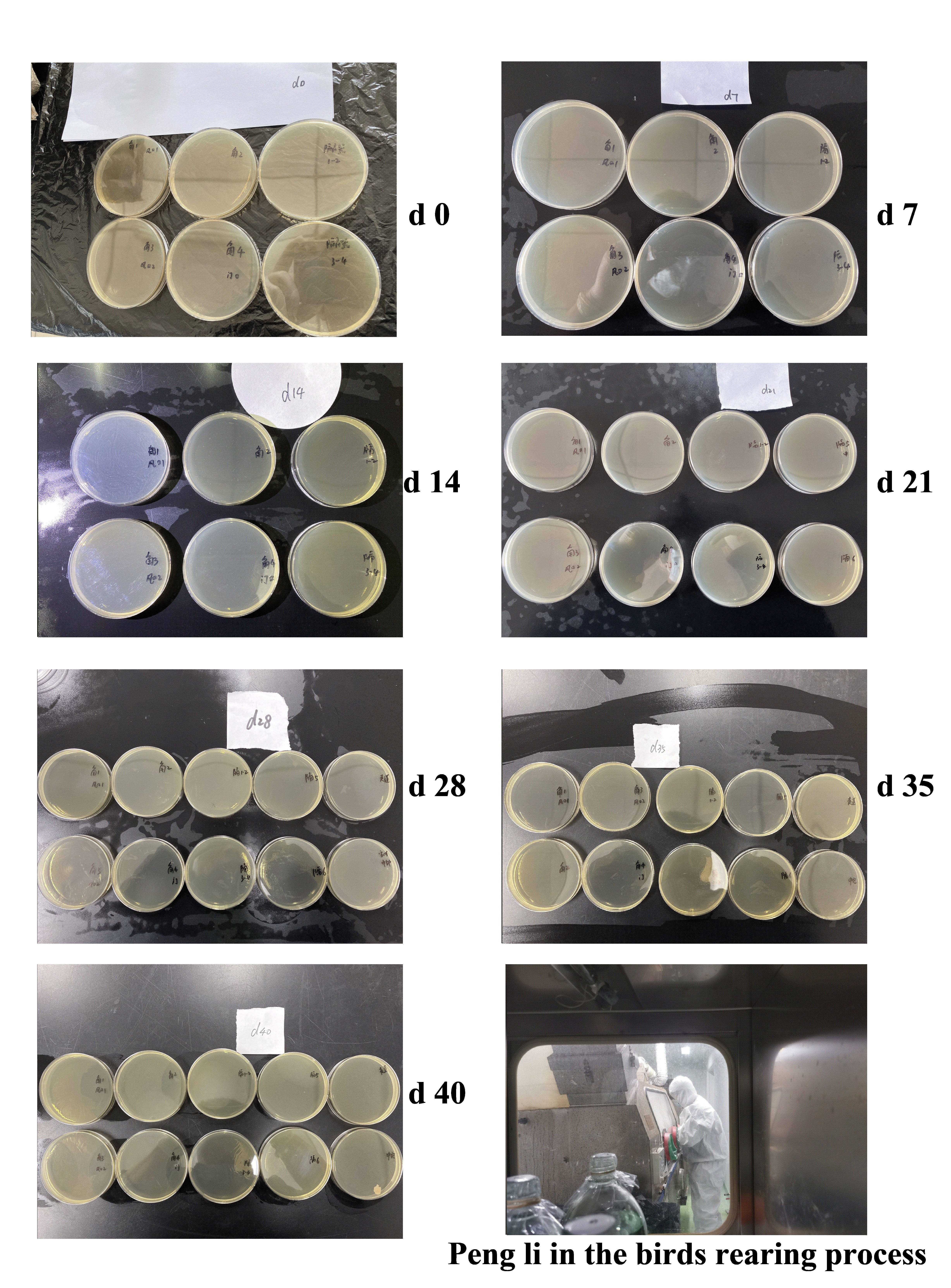

Supplement: Supplementary Figure 1 — The results of PCR and gram staining about the whole intestinal chyme mixture. According to the standard of China’s sterile animal living environment and fecal specimen testing standards (GB/T 14926.41-2001), six samples of each treatment were randomly selected for PCR analysis. The primer sequence was 27F: 5’-AGAGTTTGATCCTGGCTCAG-3’, 1492R: 5’- TACGGYTACCTTGTTACGACTT-3’. The result was shown above (A, B), and the NC represented the PCR results of RNA-free water. Then, the intestinal chyme of all birds in each treatment were mixed separately, and three samples from the mixture were randomly selected for gram stain observation. The results were shown in (C–F), among them, the gram-positive bacteria were stained purple, and the red ones represented the gram-negative bacteria. [file DataSheet_1.zip › Supplementatry Figures/Supplementatry Fig. 2.jpg]

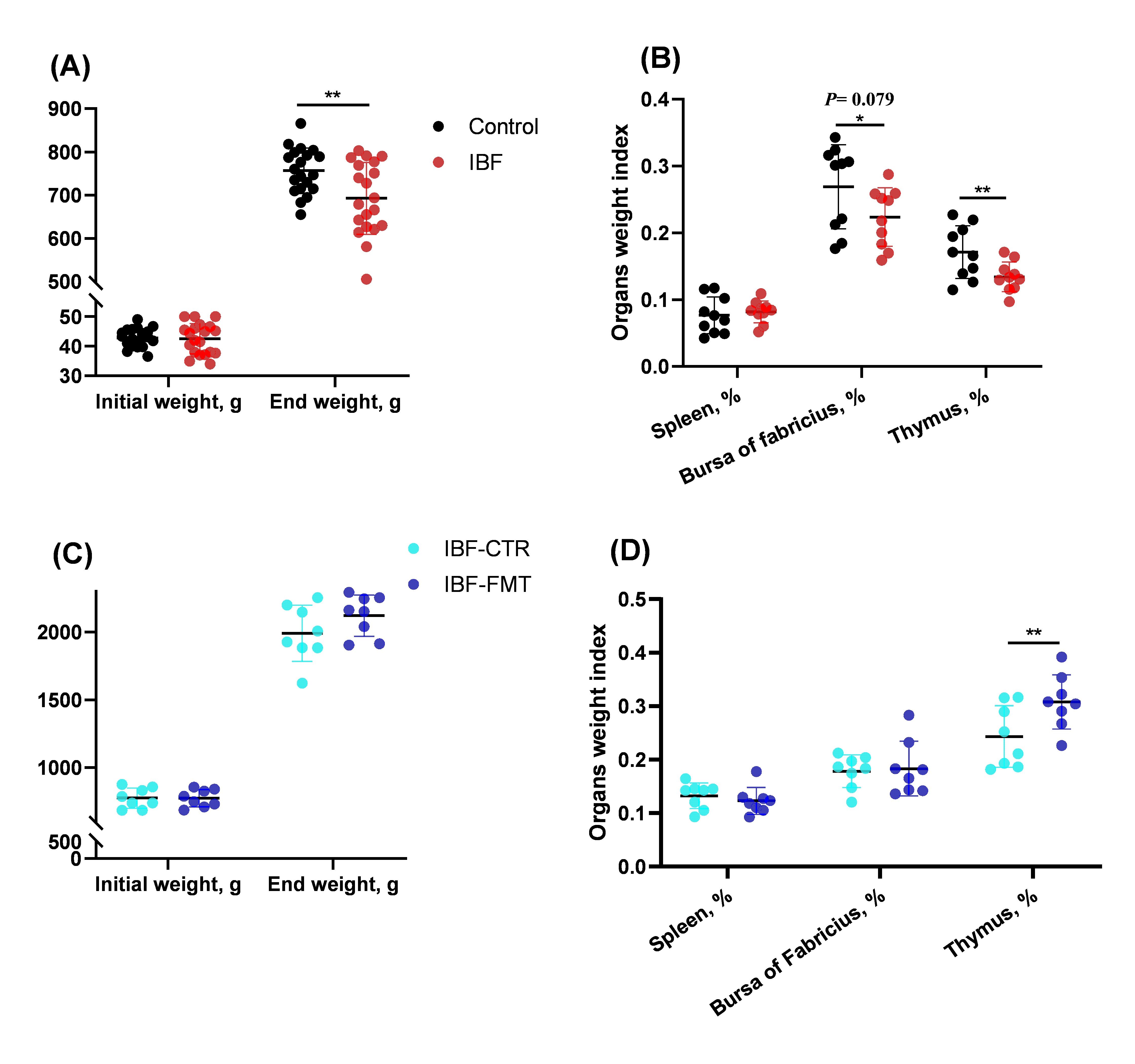

Supplement: Supplementary Figure 1 — The results of PCR and gram staining about the whole intestinal chyme mixture. According to the standard of China’s sterile animal living environment and fecal specimen testing standards (GB/T 14926.41-2001), six samples of each treatment were randomly selected for PCR analysis. The primer sequence was 27F: 5’-AGAGTTTGATCCTGGCTCAG-3’, 1492R: 5’- TACGGYTACCTTGTTACGACTT-3’. The result was shown above (A, B), and the NC represented the PCR results of RNA-free water. Then, the intestinal chyme of all birds in each treatment were mixed separately, and three samples from the mixture were randomly selected for gram stain observation. The results were shown in (C–F), among them, the gram-positive bacteria were stained purple, and the red ones represented the gram-negative bacteria. [file DataSheet_1.zip › Supplementatry Figures/Supplementatry Fig. 3.jpg]

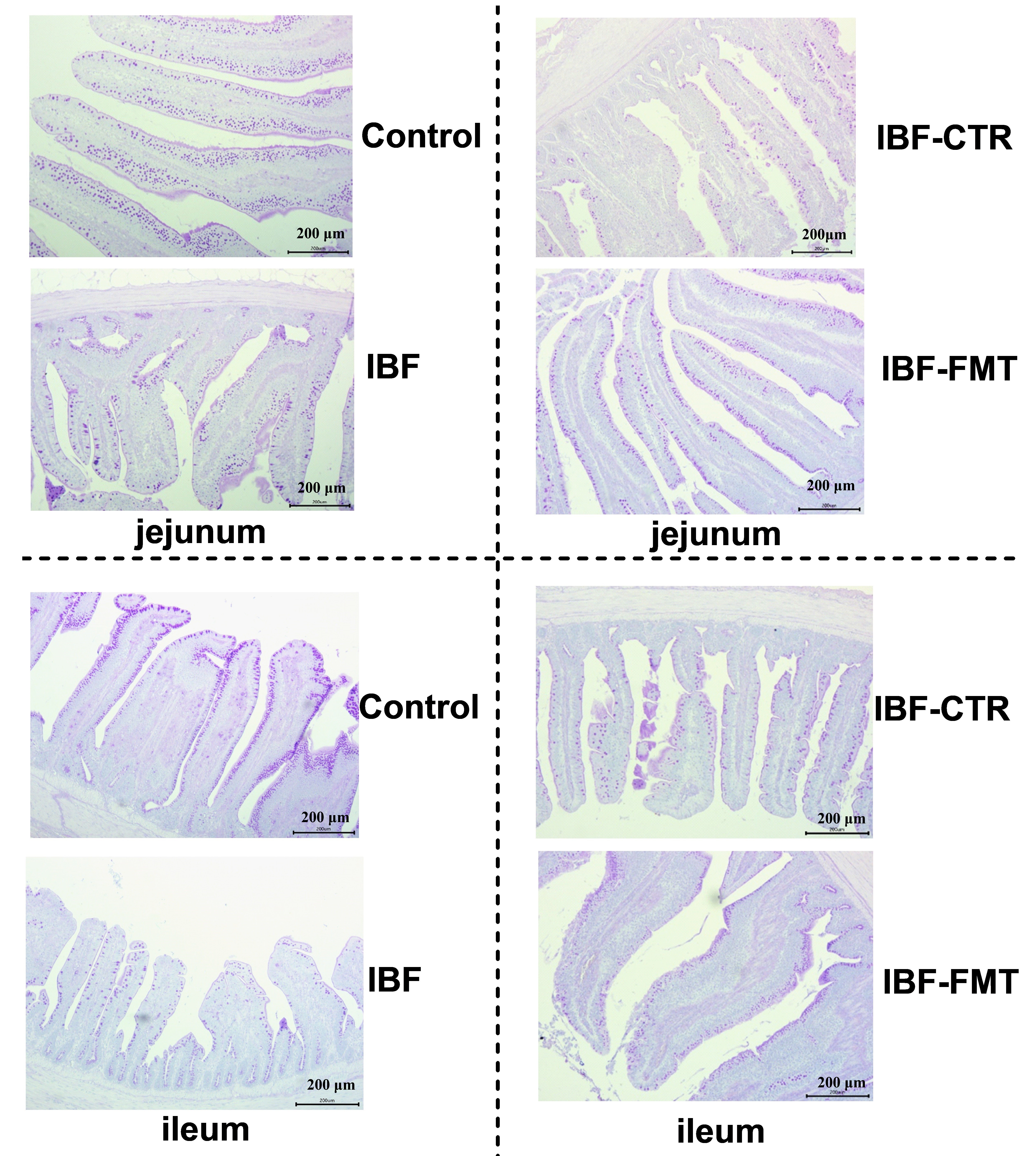

Supplement: Supplementary Figure 1 — The results of PCR and gram staining about the whole intestinal chyme mixture. According to the standard of China’s sterile animal living environment and fecal specimen testing standards (GB/T 14926.41-2001), six samples of each treatment were randomly selected for PCR analysis. The primer sequence was 27F: 5’-AGAGTTTGATCCTGGCTCAG-3’, 1492R: 5’- TACGGYTACCTTGTTACGACTT-3’. The result was shown above (A, B), and the NC represented the PCR results of RNA-free water. Then, the intestinal chyme of all birds in each treatment were mixed separately, and three samples from the mixture were randomly selected for gram stain observation. The results were shown in (C–F), among them, the gram-positive bacteria were stained purple, and the red ones represented the gram-negative bacteria. [file DataSheet_1.zip › Supplementatry Figures/Supplementatry Fig. 4.jpg]

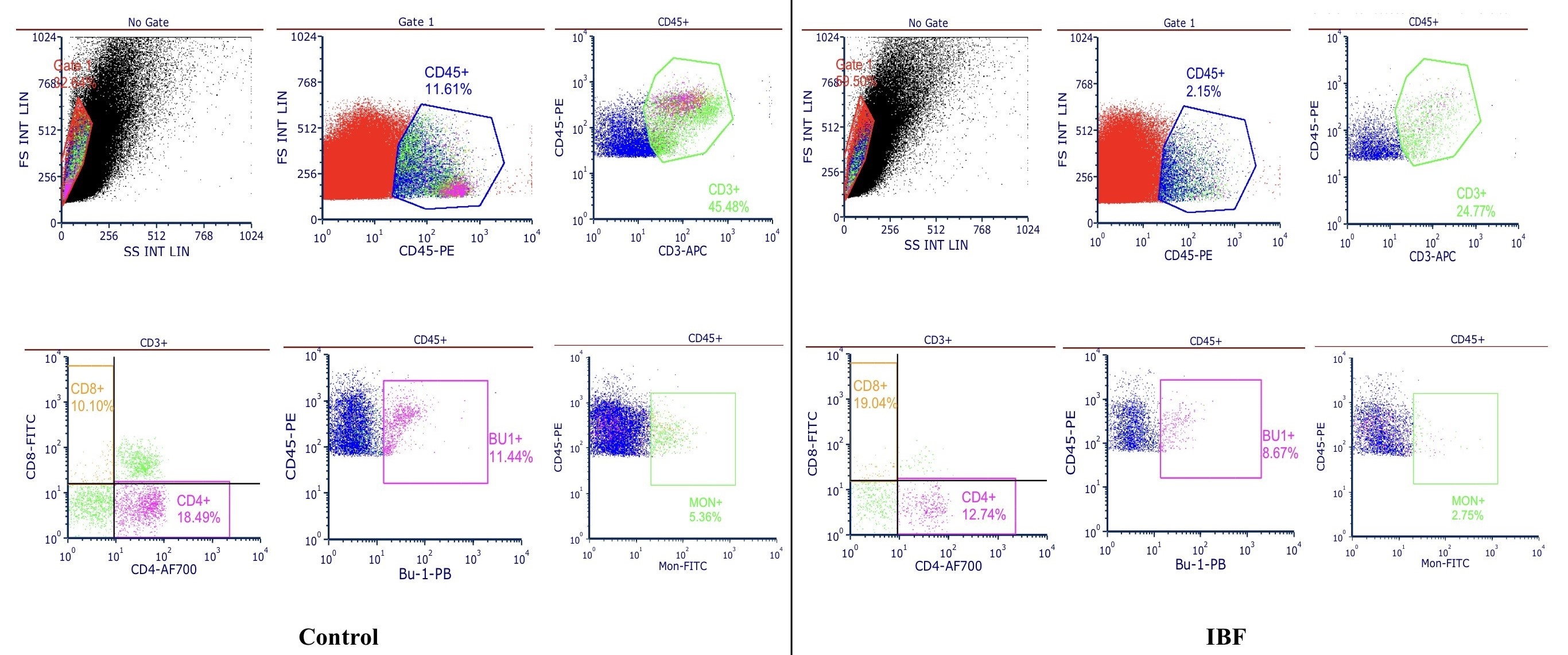

Supplement: Supplementary Figure 1 — The results of PCR and gram staining about the whole intestinal chyme mixture. According to the standard of China’s sterile animal living environment and fecal specimen testing standards (GB/T 14926.41-2001), six samples of each treatment were randomly selected for PCR analysis. The primer sequence was 27F: 5’-AGAGTTTGATCCTGGCTCAG-3’, 1492R: 5’- TACGGYTACCTTGTTACGACTT-3’. The result was shown above (A, B), and the NC represented the PCR results of RNA-free water. Then, the intestinal chyme of all birds in each treatment were mixed separately, and three samples from the mixture were randomly selected for gram stain observation. The results were shown in (C–F), among them, the gram-positive bacteria were stained purple, and the red ones represented the gram-negative bacteria. [file DataSheet_1.zip › Supplementatry Figures/Supplementatry Fig. 5.jpg]

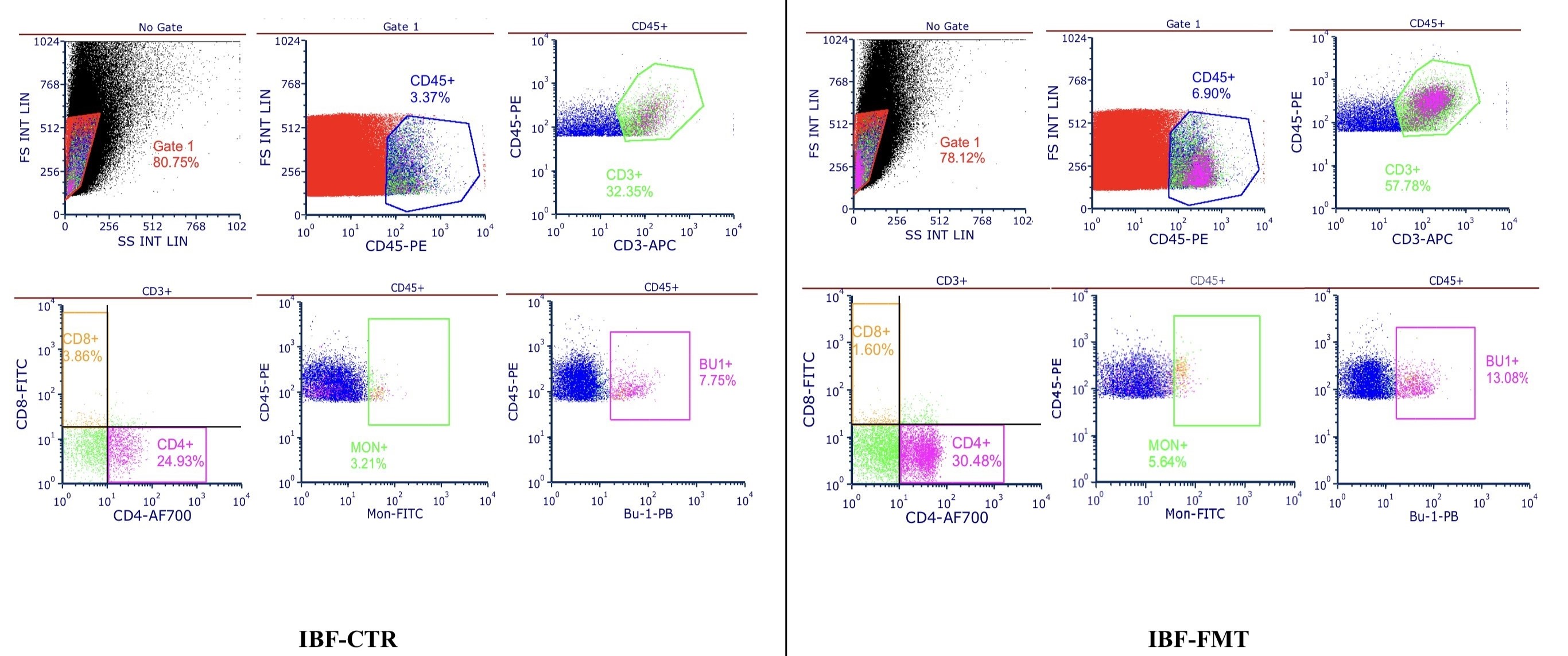

Supplement: Supplementary Figure 1 — The results of PCR and gram staining about the whole intestinal chyme mixture. According to the standard of China’s sterile animal living environment and fecal specimen testing standards (GB/T 14926.41-2001), six samples of each treatment were randomly selected for PCR analysis. The primer sequence was 27F: 5’-AGAGTTTGATCCTGGCTCAG-3’, 1492R: 5’- TACGGYTACCTTGTTACGACTT-3’. The result was shown above (A, B), and the NC represented the PCR results of RNA-free water. Then, the intestinal chyme of all birds in each treatment were mixed separately, and three samples from the mixture were randomly selected for gram stain observation. The results were shown in (C–F), among them, the gram-positive bacteria were stained purple, and the red ones represented the gram-negative bacteria. [file DataSheet_1.zip › Supplementatry Figures/Supplementatry Fig. 6.jpg]

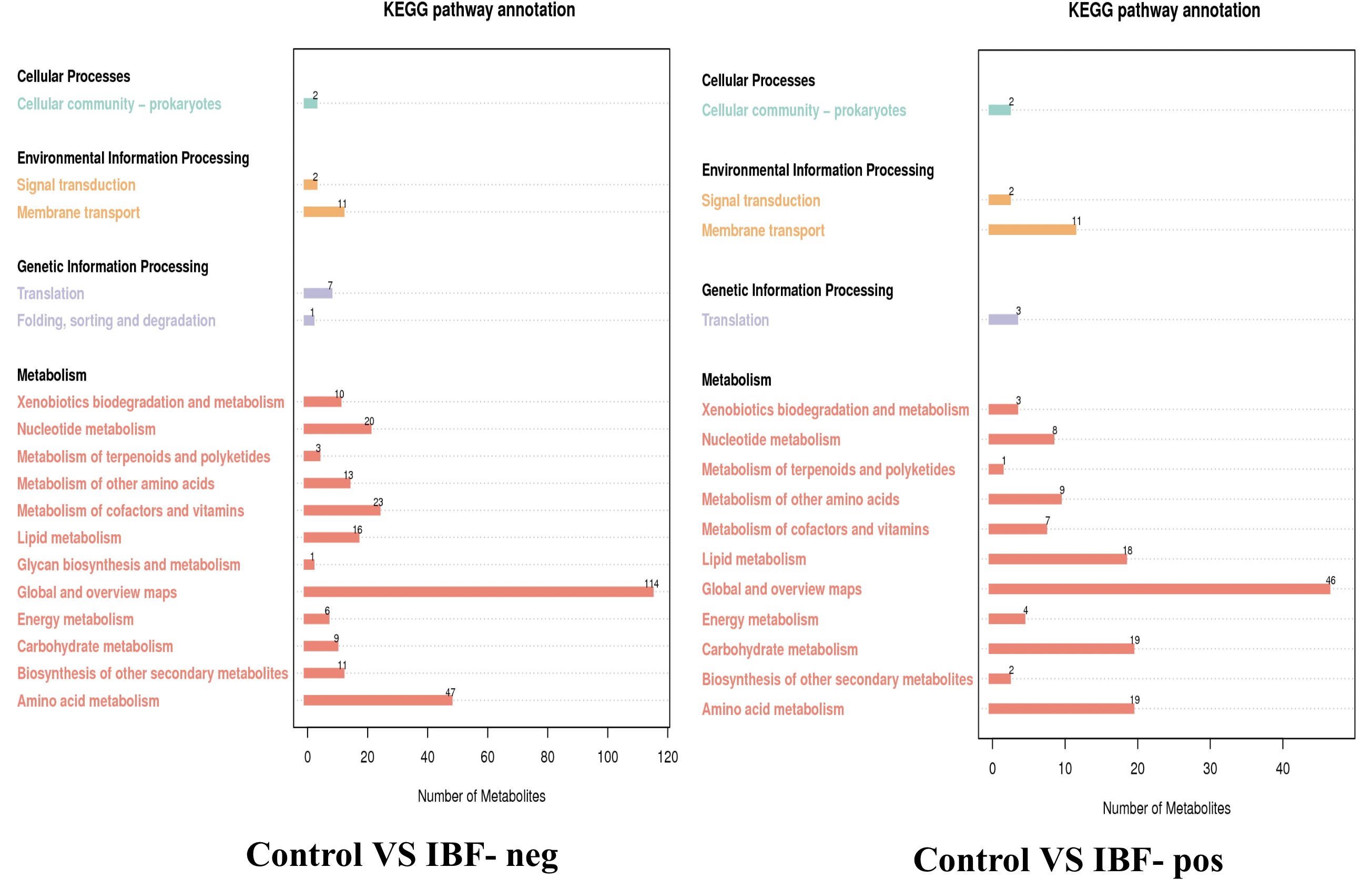

Supplement: Supplementary Figure 1 — The results of PCR and gram staining about the whole intestinal chyme mixture. According to the standard of China’s sterile animal living environment and fecal specimen testing standards (GB/T 14926.41-2001), six samples of each treatment were randomly selected for PCR analysis. The primer sequence was 27F: 5’-AGAGTTTGATCCTGGCTCAG-3’, 1492R: 5’- TACGGYTACCTTGTTACGACTT-3’. The result was shown above (A, B), and the NC represented the PCR results of RNA-free water. Then, the intestinal chyme of all birds in each treatment were mixed separately, and three samples from the mixture were randomly selected for gram stain observation. The results were shown in (C–F), among them, the gram-positive bacteria were stained purple, and the red ones represented the gram-negative bacteria. [file DataSheet_1.zip › Supplementatry Figures/Supplementatry Fig. 7.jpg]

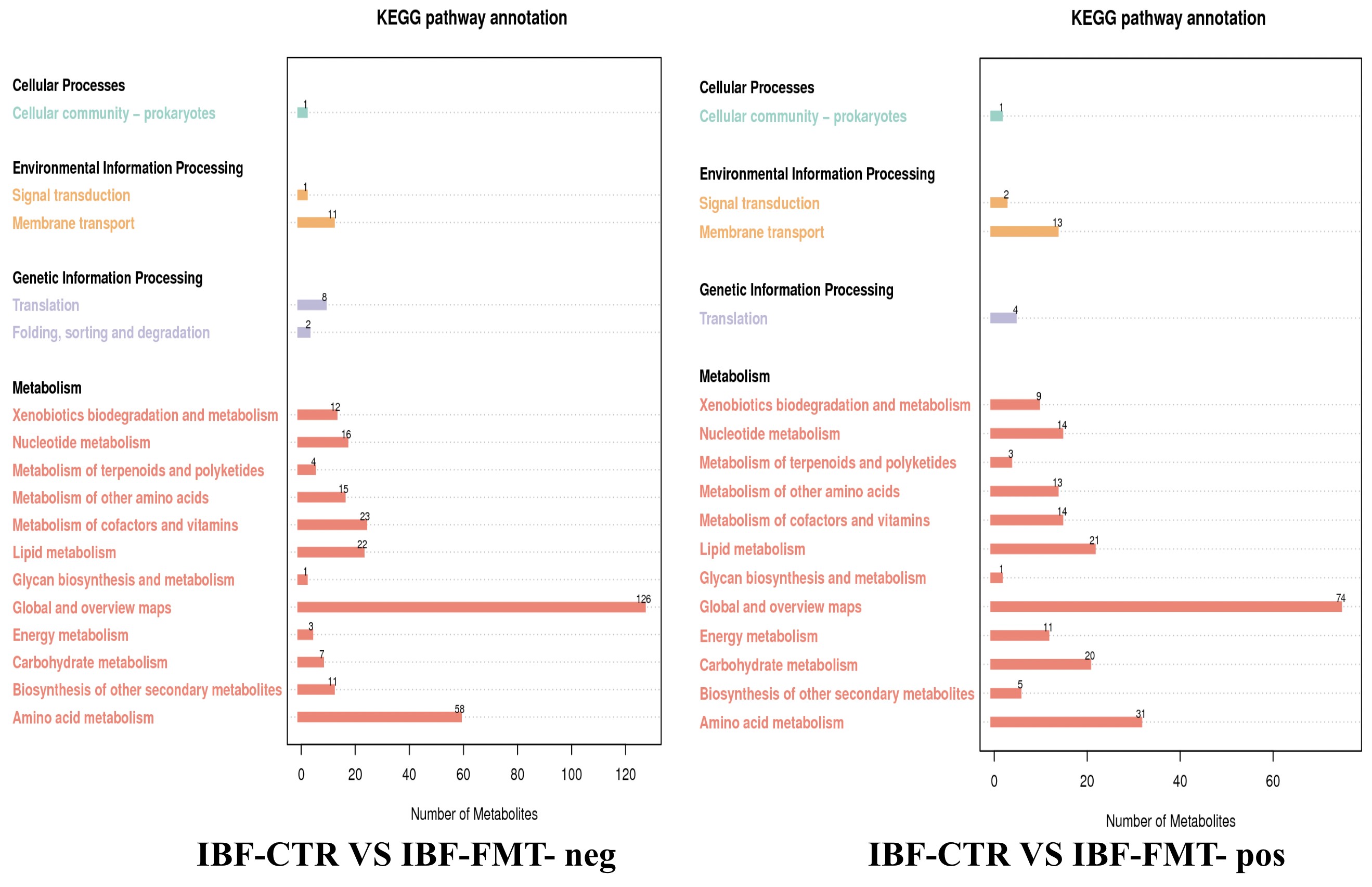

Supplement: Supplementary Figure 1 — The results of PCR and gram staining about the whole intestinal chyme mixture. According to the standard of China’s sterile animal living environment and fecal specimen testing standards (GB/T 14926.41-2001), six samples of each treatment were randomly selected for PCR analysis. The primer sequence was 27F: 5’-AGAGTTTGATCCTGGCTCAG-3’, 1492R: 5’- TACGGYTACCTTGTTACGACTT-3’. The result was shown above (A, B), and the NC represented the PCR results of RNA-free water. Then, the intestinal chyme of all birds in each treatment were mixed separately, and three samples from the mixture were randomly selected for gram stain observation. The results were shown in (C–F), among them, the gram-positive bacteria were stained purple, and the red ones represented the gram-negative bacteria. [file DataSheet_1.zip › Supplementatry Figures/Supplementatry Fig. 8.jpg]

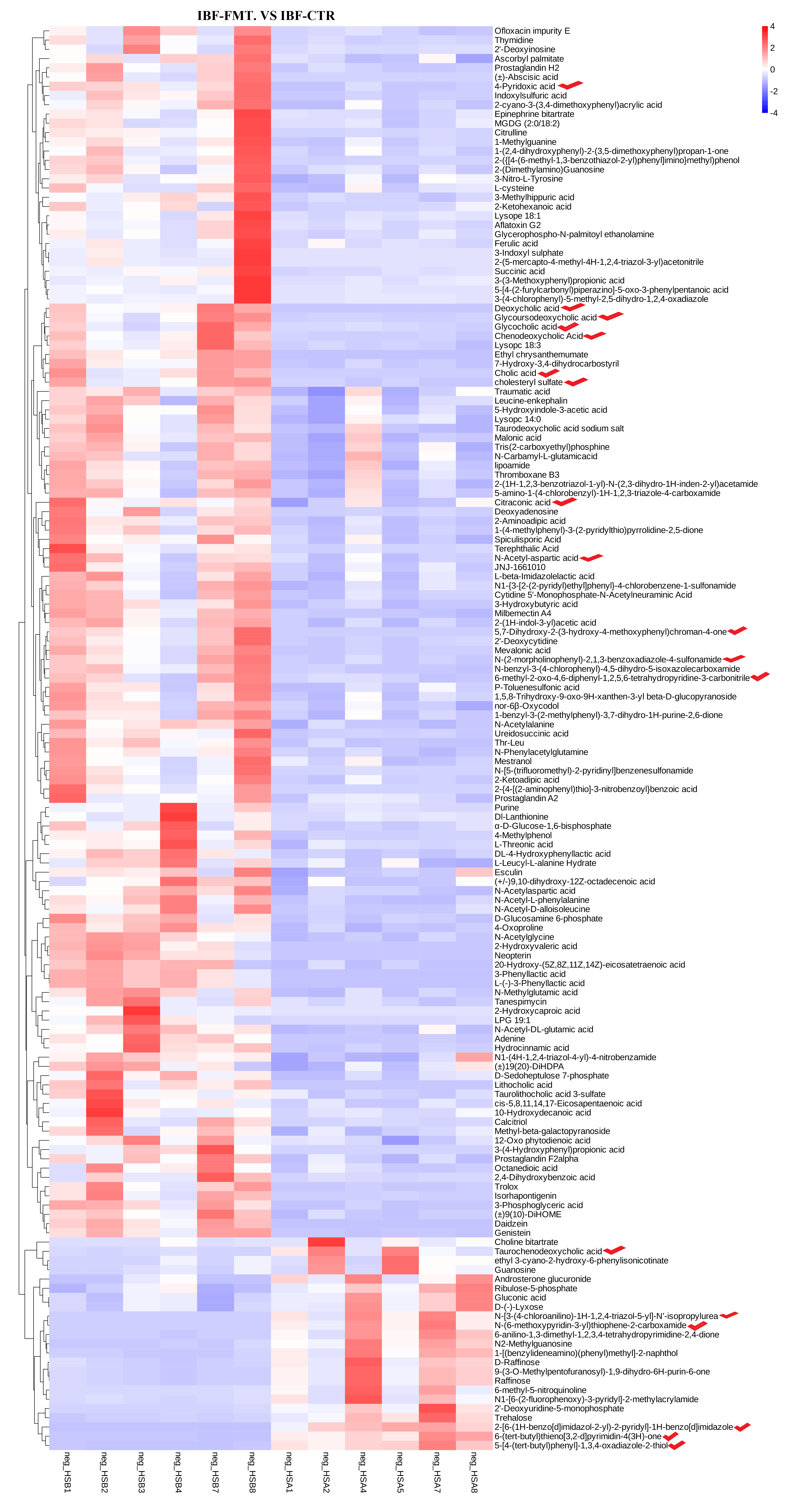

Supplement: Supplementary Figure 1 — The results of PCR and gram staining about the whole intestinal chyme mixture. According to the standard of China’s sterile animal living environment and fecal specimen testing standards (GB/T 14926.41-2001), six samples of each treatment were randomly selected for PCR analysis. The primer sequence was 27F: 5’-AGAGTTTGATCCTGGCTCAG-3’, 1492R: 5’- TACGGYTACCTTGTTACGACTT-3’. The result was shown above (A, B), and the NC represented the PCR results of RNA-free water. Then, the intestinal chyme of all birds in each treatment were mixed separately, and three samples from the mixture were randomly selected for gram stain observation. The results were shown in (C–F), among them, the gram-positive bacteria were stained purple, and the red ones represented the gram-negative bacteria. [file DataSheet_1.zip › Supplementatry Figures/Supplementatry Fig. 9.png]
